# Four New Menadione Thioderivatives, Potential Antineoplastic Candidates: In Silico and PARP-1 Inhibition Studies

**DOI:** 10.3390/molecules31060958

**Published:** 2026-03-12

**Authors:** Francisco Javier Pérez Flores, Luis Jaime Vázquez-López, Adriana Lizbeth Rivera Espejel, María Inés Nicolás-Vázquez, María Z. Saavedra-Leos, Alberto A. Fajardo de la Rosa, Samuel Álvarez-Almazán, Joel Martínez, René Miranda Ruvalcaba

**Affiliations:** 1 Laboratorio de Espectrometría de Masas, Instituto de Química, Universidad Nacional Autónoma de México, Circuito Exterior s/n, Ciudad Universitaria, Ciudad de México 04510, Mexico; jape@unam.mx; 2 Departamento de Ciencias Químicas, Facultad de Estudios Superiores Cuautitlán Campo 1, Universidad Nacional Autónoma de México, Avenida 1o de Mayo s/n, Colonia Santa María las Torres, Cuautitlán Izcalli 54740, Mexico; 315025569@cuautitlan.unam.mx (L.J.V.-L.); riveraespejeladriana@gmail.com (A.L.R.E.); nicovain@yahoo.com.mx (M.I.N.-V.); 3 Coordinación Académica Región Altiplano, Universidad Autónoma de San Luis Potosí, Carretera Cedral km, 5+600 Ejido San José de las Trojes Matehuala, San Luis Potosí 78700, Mexico; zenaida.saavedra@uaslp.mx; 4 Laboratorio de Ingeniería Química, Facultad de Química, Universidad Nacional Autónoma de México, Ciudad de México 04510, Mexico; aafdlr@ciencias.unam.mx; 5 Laboratory of Biotechnology, Unidad de Investigación Multidisciplinaria, Facultad de Estudios Superiores Cuautitlán Campus 1, Universidad Nacional Autónoma de México, Cuautitlán Izcalli 54740, Mexico; samuel.alvarez@cuautitlan.unam.mx

**Keywords:** menadione thioderivatives, olaparib, PARP-1, DFT, docking

## Abstract

The design, production, and study of new poly[ADP-ribose] polymerase 1 (PARP-1) inhibitors have emerged as an interesting exploration area, since PARP-1 is an overexpressed enzyme in several carcinomas. In this sense, menadione, or vitamin K3, is well known for its use in correct blood clotting, and for the generation of reactive oxygen species, but it is important to mention that it has been used as an antineoplastic agent against several cell lines. Related to the last commentary, in this work, four novel molecules (**2**–**5**) were produced from menadione through a Michael addition protocol, using 1,2-ethanedithiol, cysteamine, benzene-1,4-dithiol, and 4-aminobenzenethiol as nucleophiles, and menadione (**1**) as substrate, to evaluate them as plausible candidates to inhibit PARP-1. It is convenient to note that after their production and spectroscopic characterization, both docking and theoretical studies for each compound were conducted, using density functional theory (DFT) with the hybrid method B3LYP with the 6-311G(d,p) basis set. As a complement, the reactivity properties determined by DFT calculations were obtained for all compounds; the results revealed that **2** has the best properties to bind with PARP-1, and **3** offered good results. Hence, the target compounds were evaluated in vitro, determining their activity against PARP-1, using olaparib as a reference. Molecules **2** and **3** displayed the free binding energy values −7.97 and −9.35 kcal/mol, respectively, but **2** has the best IC_50_ value, 13.76 µM. It is important to highlight that **2** and **3** must be considered as potential new inhibitor agents against PARP-1, exhibiting competitive IC_50_ values with olaparib.

## 1. Introduction

Cancer is one of the main causes of death worldwide [1]. It occurs through DNA damage due to various factors such as UV radiation, reactive oxygen species, errors during replication, and cellular metabolic processes [2], contributing to tumor development and cancer cell progression [3]. Polymerase ADP-ribose 1 (PARP-1) is a protein implicated in the establishment and progression of cancer, since it plays a key role in DNA repair, recombination, and proliferation, and consequently, its inhibition in cancer cells may cause cell apoptosis [4]. PARP-1 is responsible for more than 90% of poly(ADP-ribosylation) activity [5]; in addition, it has been reported that PARP-1 is overexpressed in various carcinomas; therefore, PARP-1 inhibitors are important since they could induce death in cancer cells [6].

PARP-1 is fashioned by three domains: (1) a DNA-binding domain, (2) an auto-modification domain, and (3) a catalytic domain [7]. The last domain is highlighted because PARP-1 inhibitors could bind to the catalytic pocket, interfering with the ADP-ribosylation. In this sense, the amino acid residues His201, Tyr235, and Glu237 form the catalytic pocket necessary for the activity of PARP-1 [8]. Consequently, the design of new PARP-1 inhibitors must be a priority to target these amino acid residues.

**Figure 1:**
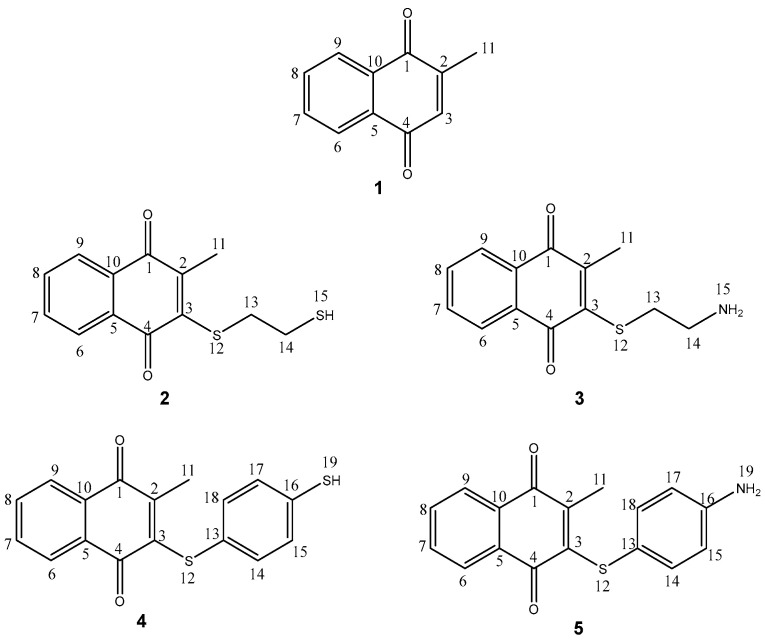
Chemical structure and proposed numeration of menadione (**1**), 2-((2-mercaptoethyl)thio)-3-methylnaphthalene-1,4-dione (**2**), 2-((2-aminoethyl)thio)-3-methylnaphthalene-1,4-dione (**3**), 2-((4-mercaptophenyl)thio)-3-methylnaphthalene-1,4-dione (**4**), and 2-((4-aminophenyl)thio)-3-methylnaphthalene-1,4-dione (**5**).

On the other hand, menadione (**1**), also known as 2-methyl-1,4-naphthoquinone or vitamin K3 (Figure 1), is a well-known chemical compound that, in general, maintains correct blood clotting and prevents excessive bleeding and hemorrhage. It is also important to note that menadione is related to the generation of reactive oxygen species that causes stress and cytotoxicity [9]. In this context, several reports have been published regarding human cervical and oral epithelial cancer cells [10], human breast cancer cell lines [11], and human hepatoma cell lines [12], among others [13]. Several works have reported different menadione derivatives supporting sulfur, oxygen, and amino substituents [13,14,15] with antineoplastic activity against specific cell lines such as MCF7 (a breast cancer cell line), highlighting that the sulfur derivative moiety bears a hydroxyl, carboxylic substituent [16], or aromatic sulfur derivatives against MCF7 and PC3 (an epithelial cell line) [13]; aromatic amino-derivatives against MCF7 have also been reported [17,18], but the formation of menadione derivatives with sulfur, oxygen, and amino atoms with direct interaction of the catalytic triad of PARP-1, to our knowledge and after a deep literature search, is scarce; thus, this is the first study which involves the interaction of new menadione derivatives with the catalytic triad of PARP-1.

As part of our research program, we have produced novel molecules with anti-neoplastic activity via PARP-1 [19,20,21,22], performing in addition the corresponding in silico studies, including comparative, theoretical, and experimental spectroscopical characterization (NMR and HRMS) [23,24,25]. Bearing in mind the previously disclosed information, the goal of this work is to obtain information related to four new menadione derivatives (**2**–**5**, Figure 1), with antineoplastic activity via PARP-1, displaying their synthesis, spectroscopical characterization and in addition using theoretical calculations employing density functional theory (DFT) and docking studies, to exhibit them as candidates for PARP-1 inhibitors.

## 2. Results and Discussion

### 2.1. Synthesis and Structural Attribution

**Scheme 1:**
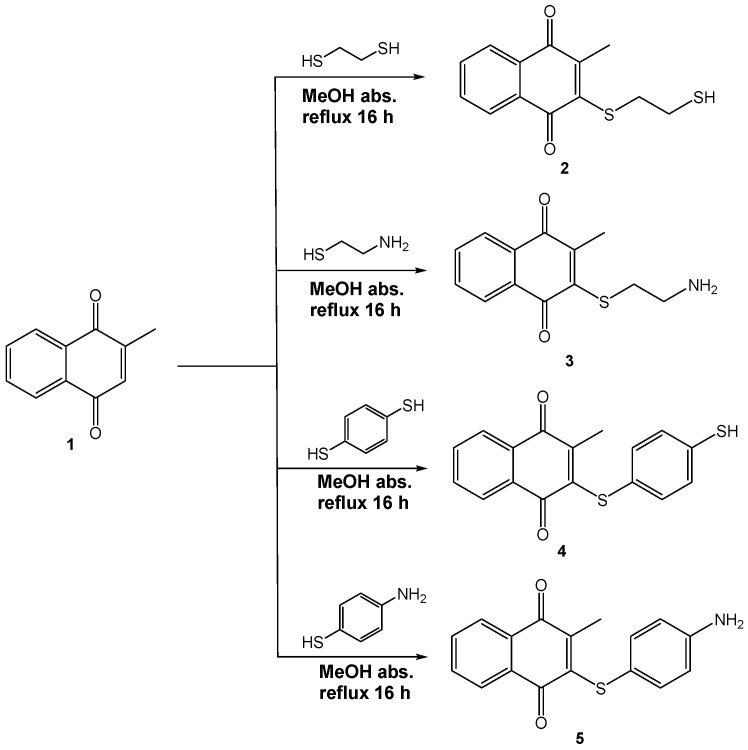
Production of menadione thioderivatives, **2**–**5**.

In the first instance, to our knowledge, after a deep literature search, the four menadione derivatives are a new set of molecules (SciFinder^®^, Scopus, and ResearchGate). Thus, to produce the target molecules **2**–**5**, **1** was mixed with 1,2-ethanedithiol, benzene-1,4-dithiol, cysteamine, or 4-aminobenzenethiol via a Michael addition protocol followed by in situ oxidation (Scheme 1). It is worth noting that the oxidative step may be promoted by the same quinone and the semiquinone of menadione by transfer of one hydrogen [26].

For the structural attribution of the obtained molecules, compound **2** is offered as a representative example: a red oil obtained with a 37% yield of pure compound. The corresponding ^1^H NMR spectrum displayed a double doublet signal in the range of 7.96–7.91 ppm, which was unequivocally assigned to the protons labeled as H9 and H6 that display an *ortho* (*J* = 8 Hz) coupling with H8 and H7, respectively. In the range of 7.76–7.75 ppm, a double-of-doublet signal was assigned to the protons H8 and H7 that have a *meta* (*J* = 4 Hz) coupling with H6 and H9, respectively.

Also, two triple signals were observed, centered at 2.95 and 2.22 ppm for H14 and H13, respectively. Due to the electron-withdrawing influence of the sulfur atom, the single signal for hydrogen supported on the sulfur atom, H15, was observed at 2.18 ppm. Finally, a single signal for H11 was detected at 2.16 ppm.

Related to the ^13^C NMR spectrum, the key signals that are consistent with the structure of **2** were assigned to the carbonyl groups at 181.76 and 180.87 ppm for C1 and C4, respectively. The signal for the methine carbon C3 was observed at 147.62 ppm. Finally, the carbons corresponding to the methylene groups C13 and C14 are highlighted at 67.41 and 38.54 ppm, respectively.

Regarding the low-resolution MS-DART^+^, the following data were displayed: an ion-fragment *m*/*z* 265(100)[M + 1]^+^ in agreement with a molecular ion protonated, in addition to a set of appropriate ion-fragments *m*/*z* 232(15)[M − 32]^+•^, *m*/*z* 231(88)[M − 33]^+^, *m*/*z* 205(43)[M − 59]^+^. It is important to highlight the HRMS-DART^+^ data by which an elemental composition of C_13_H_13_O_2_S_2_ was reached, in agreement with an exact value of 265.03570 Da correlated to a precise value of 265.03528 Da with an error of −1.56 ppm, assigned to an unequivocal ion-fragment [M + 1]^+^; complementarily, the provided unsaturation data (9.5) agrees with the structure.

### 2.2. Comparative NMR Study, Theoretical Versus Experimental Data for ***2***–***5***

**Figure 2:**
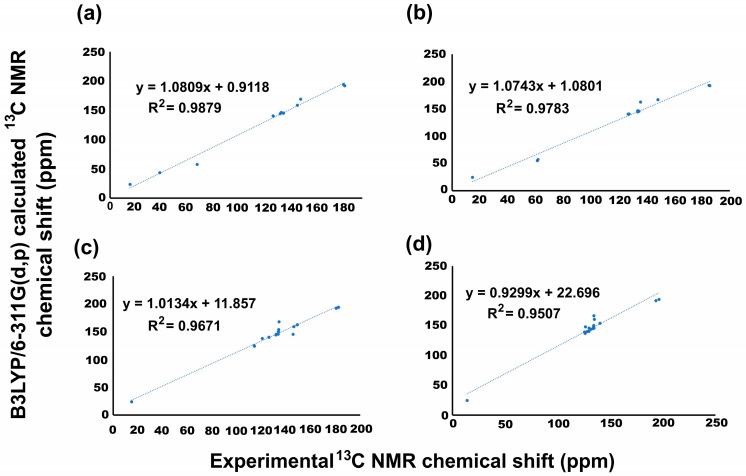
^13^C NMR linear regressions between the calculated B3LYP/6-311G(d,p) and the experimental data for (**a**) **2**, (**b**) **3**, (**c**) **4**, and (**d**) **5**.

The nuclear magnetic resonance (NMR) experimental chemical shifts for ^1^H and ^13^C for **2**–**5** were correlated with those calculated by the B3LYP level with the 6-311G(d,p) basis set and the GIAO method. In Figure 2, the corresponding correlations are displayed through linear regression; additionally, Table 1 shows both ^1^H and ^13^C NMR theoretical and experimental chemical shift values. The spectrum for all compounds can be found in the Supplementary Materials. It is important to note that for ^1^H NMR for all menadione derivatives, some difficulties in obtaining linear regression were encountered, due to overlapping experimental signals. In this sense, theoretical signals related to aromatic hydrogens can influence the regression of data. Therefore, for theoretical data, it was possible to identify the individual values, and the experimental data were expressed as a group; consequently, the linear regression was not reported.

Thus, these signals were discussed appropriately, and it is important to mention that they carry a sulfur substituent and aromatic moiety. For compounds **2** and **3**, the experimental values for H6 and H9 were observed at 7.91–7.96 and 7.93–8.02 ppm, and theoretically at 8.07 and 8.10 ppm, and 8.10 and 8.10 ppm for H6 and H9, respectively. For hydrogens labeled as H7 and H8, the experimental values were observed at 7.75–7.76 and 7.63–7.65 ppm, and the theoretical values were established at 7.48 and 7.51, and 7.51 and 7.54 ppm, respectively. Regarding H13 and H14, the experimental and theoretical values for **2** were observed at 2.22 and 2.95, and 3.23 and 2.54 ppm, respectively, and for **3** at 3.46 and 3.66, and 3.00 and 2.70 ppm. Finally, H15, the hydrogen supported on the sulfhydryl or primary amine group, was experimentally found at 2.18 and 2.50 ppm and theoretically at 1.47 and 1.11 ppm, for **2** and **3**, respectively.

For **4** and **5**, the hydrogens labeled as H6 and H9 were observed in the range of 7.62–7.64 and 7.89–7.99 ppm; the theoretical values were detected at 7.81 and 7.80 ppm for H6 and 8.07 and 8.06 ppm for H9. The experimental values for H7 and H8 were detected at 7.52–7.56 and 7.78–7.83 ppm, and the theoretical values at 7.41 and 7.38 ppm, and 7.48 and 7.43 ppm for H7 and H8, respectively. H14 and H18 were observed, for **4** and **5**, in the range of 7.40–7.41 and 7.13–7.15 ppm, and the theoretical values at 6.92 and 6.97 ppm, and 7.68 and 7.82 ppm for H14 and H18, respectively. For H15 and H17, experimental values in the range of 7.35–7.36 and 6.51–6.52 ppm were detected, and theoretical values at 6.71 and 6.17 ppm, and 6.84 and 6.40 ppm for H15 and H17, respectively.

In Figure 2, the linear regression analysis of ^13^C NMR data is shown. In this regard, the linear regression for **2** indicated a regression coefficient of 0.987949, with a standard deviation of 51.46915 and a typical error of 6.23506 ppm. The equation that describes the fit is *δ*_Theoretical_ = 1.0809*δ*_Calculated_ + 0.9118 ppm, where the slope and intercept were 1.08 and 0.91, respectively. For **3**, the regression value obtained was 0.978267, with a standard deviation and typical error of 49.96035 and 8.125054 ppm, respectively. Thus, the equation that describes the fit is *δ*_Theoretical_ = 1.0743*δ*_Calculated_ + 1.0801 ppm, where both the slope and intercept showed values of 1.08. Regarding **4**, it indicated a regression coefficient of 0.9671 with a standard deviation of 36.08711 and a typical error of 6.838699 ppm. The equation that describes the fit is *δ*_Theoretical_ = 1.0134*δ*_Calculated_ + 11.857 ppm, where the slope and intercept were 1.01 and 11.90 ppm, respectively. Finally, for **5**, the values obtained for the regression coefficient, standard deviation, and typical error were 0.9507, 36.40112, and 8.127262 ppm, respectively. In this sense, the equation that describes the fit is *δ*_Theoretical_ = 0.9299*δ*_Calculated_ + 22.696 ppm, where the slope and intercept were 0.93 and 22.7 ppm, respectively.

Additionally, a linear regression analysis of NMR data was performed considering the solvent effect (DMSO). According to Table 1, the ^1^H NMR chemical shift does not display considerable changes, and the solvent effect was not significant. On the other hand, for ^13^C NMR the regression coefficient values were obtained, being improved. Thus, for **2**, the equation that describes the fit is *δ*_Theoretical_ = 0.9601*δ*_Calculated_ + 6.2229 ppm, with a regression coefficient value of 0.9888, a standard deviation of 51.51623, and a typical error of 5.52798 ppm, where the slope and intercept showed values of 0.96 and 6.22, respectively. With regard to **3**, the regression coefficient was 0.9833, the standard deviation was 49.86011, the typical error was 6.55483 ppm, and the equation that describes the fit is *δ*_Theoretical_ = 0.9097*δ*_Calculated_ + 6.2839 ppm, with slope and intercept values of 0.90 and 6.28, respectively. For **4**, the equation that describes the fit is *δ*_Theoretical_ = 1.0156*δ*_Calculated_ − 10.205 ppm, with a regression coefficient value of 0.9495, a standard deviation of 36.08886, and a typical error of 8.62706 ppm, where the slope and intercept were 1.02 and 10.2, respectively. Finally, for **5**, the regression value obtained was 0.9767, with a standard deviation and typical error of 35.80046 and 5.58434 ppm, respectively. Thus, the equation that describes the fit is *δ*_Theoretical_ = 0.9519*δ*_Calculated_ − 1.5467 ppm, where the slope and intercept showed values of 0.95 and 1.55, respectively.

The obtained results reveal a good prediction of the chemical shifts for the NMR performed in the gas phase; however, considering the solvent (DMSO) effect for **2**, **3**, and **5**, the regression coefficient values were improved. In the case of **4**, the regression values diminished. The sulfur atom probably promoted that discrepancy. The major differences in chemical shift values were detected in carbons 2 and 3 and the aromatic ring (Table 1).

### 2.3. Bioinformatic and Biological Activity Prediction

Physicochemical and ADME-Tox properties were obtained using SwissADME (version 2017) [27], Osiris Property Explorer (version 2001) [28], and ProTox 3.0 (version 2024) to evaluate potential toxicity, pharmacokinetics [29] and Lipinski rule of five fulfillment, a methodology that includes the main parameters associated with compounds’ solubility and permeability, namely molecular weight, Log P, the number of H-bond donors (nOHNH), the number of H-bond acceptors (nON), and the topological polar surface area (TPSA) [30] (Table 2).

**Figure 3:**
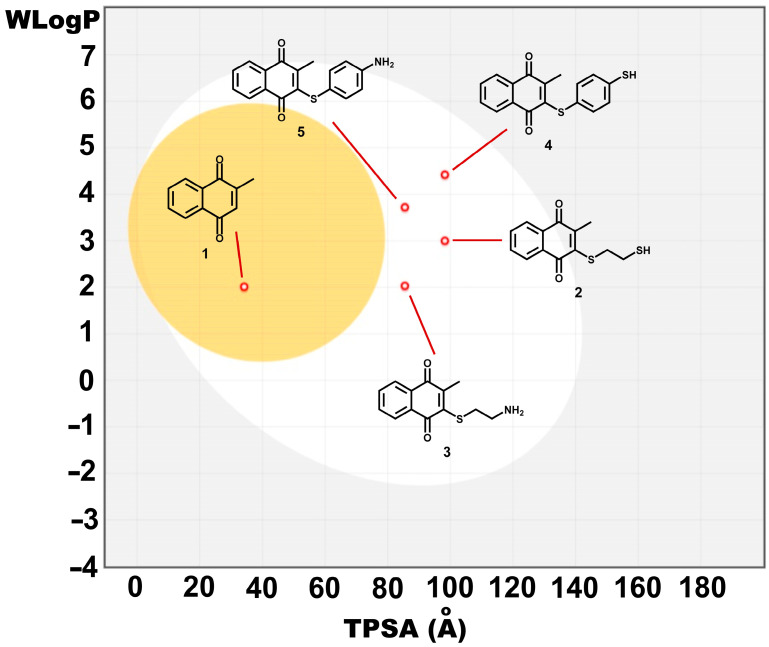
Boiled egg representation for compounds **1**–**5**.

Table 2 shows a comparison among physicochemical predicted properties for **1**–**5**. No violations of Lipinski’s rule of five were noted. All compounds showed no more than five H-bond donors (nOHNH) or ten H-bond acceptors (nON) and a molecular weight of no greater than 500 g/mol, suggesting possible better absorption and permeation. MLogP preferred values are lower than 4.15; compounds **4** and **5** exhibited greater MLogP values (3.07 and 2.19, respectively), due to the additional benzene ring in benzene-1,4-dithiol and 4-aminobenzenethiol [30]. MLogP values are also related to high gastrointestinal absorption for all compounds, favorable when oral administration is desired. Figure 3 shows a boiled egg representation, in which the lipophilicity and polarity of small molecules are computed, viewing the white zone as the possibility to be passively absorbed by the gastrointestinal tract and the yellow zone as the location where molecules can permeate through the blood–brain barrier [33]. In this case, only **1** displayed possibility to pass through the blood–brain barrier owing to the lowest TPSA value and its small molecular weight.

On the other hand, considering further pharmacokinetic parameters (Table 3), none of the compounds are P-glycoprotein (P-gp) substrates; however, all of them could be inhibitors of at least one of the most important CYP450 isoforms. Additionally, toxicity prediction showed that proposed modifications to **1** decreased toxicity risks for **2**–**5**, including mutagenic, tumorigenic, and reproductive effect risk. A drug score was obtained from Osiris Property Explorer based on the combination of three physicochemical properties (logP, logS, and molecular weight) and toxicity property predictions, aiming to achieve the closest score to 1 [28]. The best drug score was found with **3** and **5** (0.55 and 0.48, respectively).

The toxicity study was complemented with a toxicity class prediction with ProTox 3.0, employing a color coding from 1 to 6, where toxicity class 6 is the most desired and toxicity class 1 is the least desired toxicity class. Compounds **4** and **5** showed a class 4 toxicity prediction, while **2** and **3** displayed toxicity class 3.

### 2.4. Docking Studies of ***2***–***5***

**Figure 4:**
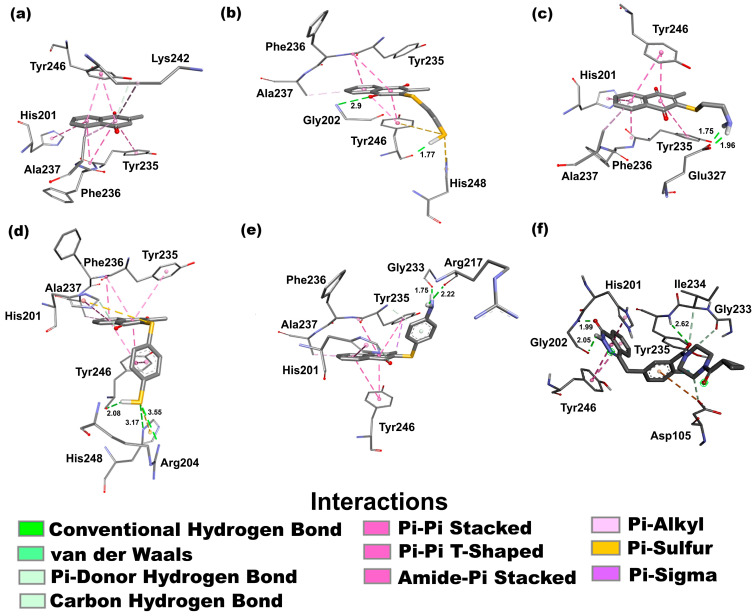
Amino acid residue interactions between PARP-1 and menadione derivatives: (**a**) menadione, **1**; (**b**) **2**; (**c**) **3**; (**d**) **4**; (**e**) **5**; and (**f**) olaparib.

Docking studies represent a computational tool for calculating, at an atomic level, the most favorable position of interaction between a protein and its ligand [34], in particular, compounds **2**–**5** (ligands) with the enzyme PARP-1, generating a three-dimensional representation. It is worth noting that several docking studies involving proteins in the apoptosis pathway have been reported elsewhere [19,20,21,22,35]. In this sense, the interaction energy (ΔG) obtained for compounds **2**–**5** was −7.97, −9.35, −9.85, and −9.85 kcal/mol, respectively. Additionally, the ΔG of menadione (**1**) and olaparib, employed as a reference, was calculated, displaying values of −7.13 and −10.23 kcal/mol, respectively. The interactions established with PARP-1 in the complex with the lowest affinity and the highest frequency are shown in Figure 4 and described below.

The ligands that have the best stability of interaction with the protein are those that have a lower energy requirement. In this context, considering the reference, the order of stability was olaparib > **4** = **5** > **3** > **2** > **1**. It is important to note that these compounds interact with at least two amino acid residues of the catalytic triad.

Figure 4a displays the interactions of **1** with PARP-1 through a π-hydrogen bond donor between the OH group of Tyr246 and the quinonic system. Also, π-π stacked interactions are shown between aromatic and carbonyl groups of Tyr235 and quinonic and aromatic systems, the imidazole ring of His201 and the aromatic ring, and the aromatic ring of Tyr246 and quinonic and aromatic systems. Further, one π–alkyl or hydrophobic interaction between the methyl group of Ala237 and quinonic and aromatic rings is displayed.

For **2** (Figure 4b), two hydrogen bond interactions are displayed between the carbonyl group of Tyr246 and the hydrogen of the sulfhydryl group (1.77 Å), and another between the carbonyl of the quinonic system and the hydrogen of the amino group of Gly202 (2.9 Å), essential for the inhibitory activity [36]. Also, two π–sulfur interactions are shown between the imidazole ring of His248 and the sulfhydryl group, and between the aromatic ring of Tyr246 and the sulfhydryl group. Moreover, two π-π stacked interactions between the aromatic ring of Tyr246 and both aromatic and quinonic rings of **2** are shown. Furthermore, the amide bond generated by the union of Phe236 and Tyr235 amino acid residues exhibits an amide–π interaction with quinonic and aromatic rings. Finally, a hydrophobic interaction type was observed between the methyl group of Ala237 and the aromatic ring of **2**.

For **3**, in Figure 4c, two hydrogen bond interactions can be seen with the amino acid residue Glu327, generated with the terminal carboxylic group of the amino acid with the hydrogens of the amine group (1.96 and 1.75 Å, respectively). Also, four π-π stacked interactions are shown between Tyr246 and the aromatic ring, and the quinonic system and the aromatic ring, in addition to the aromatic moiety of Tyr235 with the quinonic system, and the imidazole ring of His201 with the aromatic ring. Furthermore, the amide bond generated by the union of Phe236 and Tyr235 amino acid residues exhibits an amide–π interaction with the aromatic ring moiety of **3**. Finally, a hydrophobic interaction was observed between the methyl group of Ala237 and the aromatic ring moiety of **3**.

Figure 4d exhibits, for compound **4**, three hydrogen bond interactions between the sulfhydryl group and the carbonyl group of Tyr246 (2.08 Å), the NH group supported in the imidazole moiety of His248 (3.17 Å), and the NH_2_ group of the guanidine moiety of amino acid residue Arg204 (3.55 Å). Two π–sulfur interactions are exhibited between the imidazole moiety of His201 and the sulfur atom, and between the imidazole moiety of His248 and the sulfhydryl group of **4**. Five π-π stacked interactions were detected between the aromatic ring of Tyr246 and the aromatic and quinonic systems, in addition to the aromatic ring of the substituent of **4**, the aromatic ring of Tyr235 and the quinonic system, and the imidazole moiety of His201 and the aromatic ring. Also, one carbon–hydrogen bond was observed between the carbon located between the nitrogen atoms of His201 with the carbonyl group adjacent to the carbon that bears a sulfur atom. The amide bond generated by the union of Phe236 and Tyr235 amino acid residues exhibits an amide–π interaction with the aromatic ring of **4**. Finally, a hydrophobic interaction was observed between the methyl group of Ala237 and the aromatic ring moiety of **4**.

For **5**, Figure 4e shows two hydrogen bond interactions: the carbonyl group of amino acid residues Arg217 (2.22 Å) and Gly233 (1.75 Å) interacts with the hydrogens of the amine group. A π–donor hydrogen interaction was observed between the NH_2_ group of Tyr 235 and the aromatic ring of the substituent of **5**. Four π-π stacked interactions were observed between the aromatic ring of amino acid residues Tyr246 and Tyr235 with quinonic and aromatic systems, and quinonic system, respectively. Moreover, an interaction between the imidazole moiety of amino acid residue His201 and the aromatic ring of **5** was observed. Finally, two hydrophobic interactions were observed: the first between the aromatic group of amino acid residue Tyr235 and the methyl group of **5**, and the second between the methyl group of amino acid residue Ala237 and the aromatic ring of **5**.

With regard to olaparib (Figure 4f), it is important to highlight that it interacts with two amino acid residues of the catalytic triad, Tyr235 by hydrogen bond (2.62 Å) and with the amino acid residue His201 by π-π stacked interaction, but it is interesting to note that olaparib maintains two hydrogen bonds with Gly202 (1.99 and 2.05 Å) and two π-π stacked interaction with the amino acid residue Tyr246, amino acid residues key for inhibitory activity [36]. Additionally, the residue Tyr235 forms a peptidic bond with other amino acid residues such as Ile234 and Gly233, revealing other interaction types: carbon–hydrogen bonds and an anion-π interaction with the amino acid residue Asp105.

According to the structures of the menadione derivatives, **2**–**5**, all of them display higher free binding energy (−7.97 to −9.85 kcal/mol) in comparison with olaparib (−10.23 kcal/mol), but it is important to highlight that all of them bear a group (-NH_2_ or -SH) that generated hydrogens bonds. In general, all derivatives displayed interactions with two amino acid residues of the catalytic triad of PARP-1, Tyr235 and His201. In this sense, the main π-π stacked interactions were observed with Tyr235 and His201 for all compounds. In addition, for **3**, it is important to highlight that this derivative maintains interaction with the amino acid Glu327; in consequence, two hydrogen bonds were observed between the terminal carboxylic group and the hydrogens of the amine group. With regard to **2**, it maintains interaction with amino acid residues Tyr246 and Gly202, which are important to inhibitory activity [36]; the interaction with Gly202 was observed only in olaparib. Thus, it is logical to suppose that **2** and **3** could display better inhibitory activity against PARP-1; see below.

### 2.5. PARP-1 Inhibition Study

It is well known that the major inhibitor of the PARP-1 enzyme is olaparib [19,20]; therefore, the IC_50_ for olaparib was determined for this study. In this sense, **2** and **3** display moderate PARP-1 inhibitory activity with an IC_50_ of 13.76 and 14.38 µM, respectively (Table 4).

Considering the above commentaries, it is important to mention that with the addition of an alkyl moiety to groups that form hydrogen bonds to menadione, the π-π interactions and hydrogen bond were improved; see above. Thus, the concentration to inhibit PARP-1 diminished. Consequently, **2** and **3** must be considered as potential new inhibitor agents against PARP-1 due to exhibiting a moderate IC_50_ compared to olaparib. Additionally, compounds **2** and **3** do not display mutagenic, tumorigenic, or irritating risk and show good gastrointestinal absorption (Table 3). It is important to highlight that not only the catalytic triad should be considered; the amino acid residues Tyr246, Gly202, and Ser243 must also be considered [36].

### 2.6. Quantum Chemistry Studies

#### 2.6.1. DFT Optimization of **2**–**5**

Molecules **2**–**5** were optimized using the B3LYP/6-311G(d,p) level of density functional theory in Gaussian 16. Vibrational frequencies were calculated on geometry-optimized structures using an analytical Hessian program, and the character of the ground states was confirmed by non-imaginary frequency calculations, performed at the same level.

**Figure 5:**
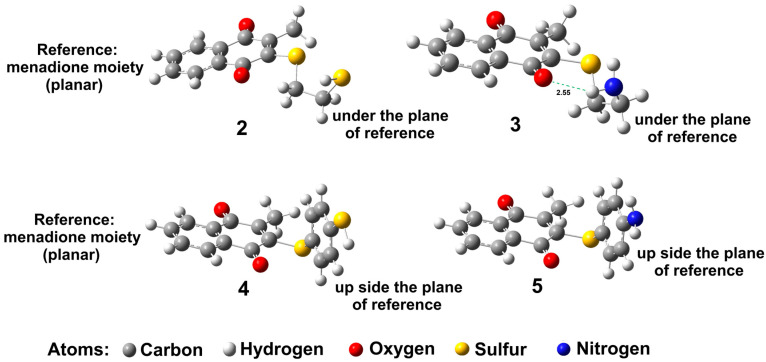
Optimized geometry of the most stable conformers for menadione thioderivatives **2**–**5**.

Additionally, the menadione moiety was considered as a reference point; this moiety is planar, and the substituent in **2** and **3** is positioned under this plane. It also can be observed that for these molecules the sulfide and amino substituents adopt a conformation-like *cis*, but for **3** the amino group is oriented toward the oxygen of the carbonyl group to form an intramolecular hydrogen bond, 2.55 Å (Figure 5).

With regard to compounds **4** and **5**, it can be observed that the substituents are positioned on the upper side of the plane of menadione, and the aromatic ring of the substituents is placed perpendicular to this plane.

Moreover, comparing the optimized structures (Figure 5) with the structures obtained in the docking studies, it can be observed that for molecule **2**, the substituent displays an extended form (Figure 4b). The molecule also shows a hydrogen bond with the oxygen atom of quinonic system; this extended form probably facilitates the double π–sulfur interaction and increases the inhibition activity against PARP-1 (Table 4).

#### 2.6.2. Geometrical Parameters for **2**–**5**

Selected structural parameters for optimized menadione derivatives **2**–**5** were calculated using density functional theory (DFT), with the B3LYP/6-311G(d,p) method. The bond lengths in Å and bond angles in degrees are confined in Table 5 and Table 6, respectively. The numeration follows Figure 1.

For this section, the bond lengths were compared with values previously reported for menadione (**1**) using X-ray powder diffraction [38] and single-crystal X-ray diffraction [39]. In this sense, it can be observed that the distances of the menadione skeleton exhibit small changes in length, principally in the quinonic ring, in contrast with experimental values reported previously for menadione. Consequently, the discussion was focused on the principal changes. Thus, the C3-C4 bond ($\bar{X}$ = 1.501 Å) was observed, which is the closest bond to the sulfur atom. The elongation of these bonds can be explained by the resonance effect of the lone electron pair at the sulfur atom through the double bond of C2-C3 toward the oxygen atom of the C1=O bond [23]. With regard to C3-S12, for molecules **2** and **3**, a bond elongation of 1.775 and 1.776 Å, respectively, was observed, which was like other quinones previously reported with values of $\bar{X}$ = 1.766 and 1.774 Å [23]. For compounds **4** and **5**, the bond elongation was slightly higher, 1.783 and 1.784 Å, respectively, probably due to a major steric hindrance, because the aromatic ring is perpendicular to the menadione moiety, as shown in Figure 5.

Considering the above commentaries, for compounds **2**–**5**, only the ring that supports the α,β-unsaturated system was selected to discuss the bond angles in comparison with the experimental data of menadione [31,32], since the major changes were perceived in this moiety due to the substitution carried out at C3.

Table 6 displays the bond angle values for **2**–**5**; the slightly higher distances are caused by the steric effect of the sulfide atom. In this sense, the bond length C1-C2-C11 was slightly short in comparison with the experimental data (116.345 degrees), possibly because the sulfur atom pushed the methyl group toward the oxygen atom. The bond angles C2-C3-S12 (118.857 and 118.926 degrees, respectively) and C3-S12-C13 (105.105 and 106.515 degrees, respectively) for compounds **2** and **3** are in concordance with the hybridization of the carbon sp^2^ and sp^3^, respectively. On the other hand, for molecules **4** and **5**, the bond angles C2-C3-S12 (121.647 and 120.044 degrees, respectively) and C3-S12-C13 (120.636 and 120.319 degrees, respectively) correspond to sp^2^ hybridization. Additionally, for the bond angle S12-C3-C4, the low values are probably a consequence of the π-π interaction between the aromatic ring of the substituent and the double bond C4=O.

#### 2.6.3. Frontier Molecular Orbital (FMO: HOMO-LUMO) Parameters for **2**–**5**

**Figure 6:**
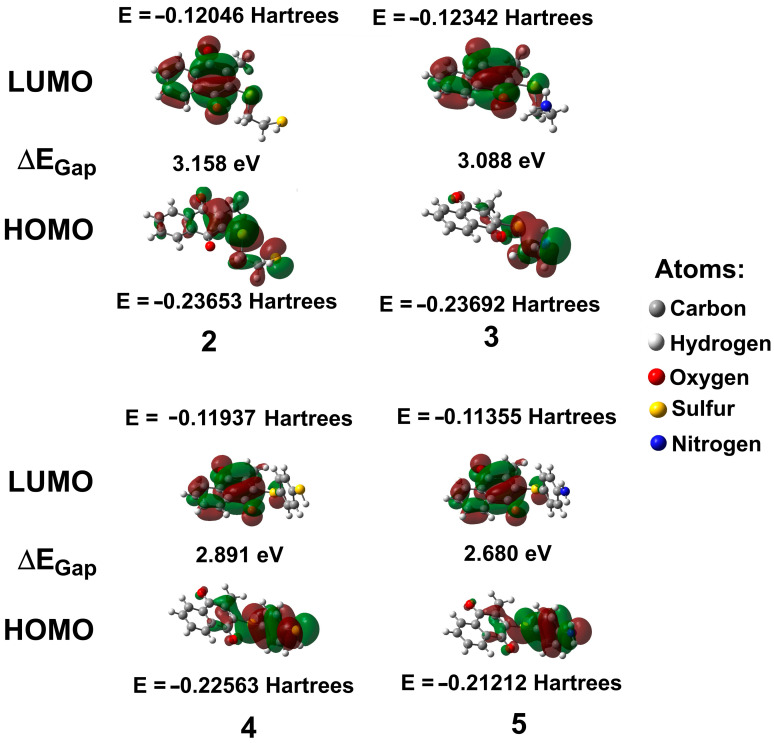
Frontier orbital HOMO-LUMO of molecules **2**–**5**.

Figure 6 shows the results obtained from the energies of the frontier orbitals, in the gas phase, for **2**–**5**: the highest occupied molecular orbital (HOMO) and the lowest unoccupied molecular orbital (LUMO). The first represents the region that donates electrons, while the second represents the region that accepts electrons; additionally, the band gap energy (ΔE_Gap_), electronegativity (χ), chemical potential (µ), electrophilicity index (ω), ionization potential (IP), electron affinity (EA), and chemical hardness (η) were calculated (Table 7) [40]. These parameters can be used to predict the biological reactivity and the chemical stability of the systems.

The HOMO for all compounds was located principally in the substituents and to a lesser extent in the double bond of the quinonic ring, except for **2**, where the HOMO also has a contribution from the oxygen of the carbonyl that displays conjugation with the double bond. Consequently, these moieties can be considered nucleophilic centers and may contribute to activity against PARP-1. On the other hand, the LUMO, in general, was in the naphthoquinone moiety.

#### 2.6.4. Reactivity Properties of **2**–**5**

The IP and EA for **2**–**5** changed as **3** > **2** > **4** > **5**; in this context, all molecules gain and lose electrons. According to the ΔE_Gap_ values, the interactions for **4**–**5** are slightly higher than **3** and **2** because the ΔE_LUMO-HOMO_ for the compounds was obtained as **2** (3.158 eV) > **3** (3.088 eV) > **4** (2.891 eV) > **5** (2.680 eV); in consequence, it can be assumed that **3** and **2** are harder (3.23 and 3.20 eV, respectively) in comparison with **4**–**5** in gas phase. In addition, the ω values for **2**–**5** are indicative of their acidic features (Table 7). Furthermore, it was observed that **3** has a higher electrophilic character (3.78 eV), indicating that it has a slightly higher capability for charge transfer than the other compounds. Regarding µ, the highest values are displayed for **3** and **2** (−4.98 and −4.84 eV, respectively), where this property may be associated with the nucleophilic character of the molecule being more reactive than **4** and **5**. In addition, the highest χ values for **3** and **2** (4.98 and 4.84 eV, respectively) indicate that both are capable of attracting electrons to themselves. Consequently, the nucleophilicity of **3** and **2** may be the reason for their inhibitory activity against PARP-1, as shown in Table 4. Finally, it is worth highlighting that all compounds possess the capacity to act as both a hydrogen donor and acceptor, a feature particularly characteristic of **1** [38].

#### 2.6.5. Molecular Electrostatic Potential (MEP) of **2**–**5**

**Figure 7:**
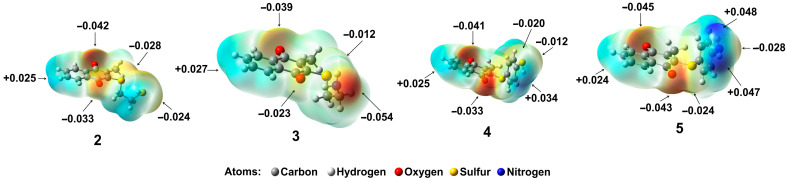
Molecular electrostatic potential in eV for **2**–**5**.

The MEP plots indicate the electrophilic and nucleophilic sites. The blue color is used to describe a positive potential, which establishes the electrophilic center, while the negative potential is visualized in red, representing the nucleophilic attack center. In Figure 7, the MEP plot for **2**–**5** shows that the negative potentials are centered at the oxygen atoms of the carbonyl groups and the nitrogen atom due to lone electron pairs. Thus, the higher electron density is placed on molecule **3**, −0.054 eV, with a positive potential value of +0.054 kcal/mol. Then, compound **5** displays a negative potential value of −0.04825 eV and a positive potential value of +0.04825 eV. Afterward, compound **2** displays negative and positive potential values of −0.0423 and +0.0423 eV, respectively. Finally, compound **4** shows the lower electron density, −0.0423 eV, with a positive potential value of +0.0423 eV, with the positive potential regions mainly being over the hydrogen atoms and hydrogen of amino or sulfhydryl groups.

According to the MEP, the positive (blue) and negative (red) sites could be indicative of the possible formation of hydrophobic, π-π, or hydrogen bond interactions between the ligand and amino acid residues of PARP-1. Thus, due to a high electron density and a higher MEP value of **2** and **3**, these features contributed to the development of interactions with amino acids, highlighting the two hydrogen bonds described above and resulting in good inhibition of PARP-1; see Table 4.

In contrast, **4**, which shows a low-density zone, displays poor interactions with amino acids, highlighting the five π-π interactions and diminishing the PARP-1 inhibition. Also, it is important to highlight compound **2**, because it shows a similar density zone to **4**. But it is necessary to note that **2** is the only compound that displays two hydrogen bonds, between amino acid residue Gly202 and the carbonyl group of the quinonic system, and between amino acid residue Tyr246 and the hydrogen of SH group; in addition, there are π–sulfur interactions between Tyr246 and the same group, and two π-π stacked interactions between Tyr246 and the naphthoquinone ring (Figure 4b); consequently, these amino acid have been considered essential to inhibit PARP-1 together with the catalytic triad [36], which may be the reason for the better inhibitory activity of **2** in comparison to **3** on PARP-1.

#### 2.6.6. Atomic Charges of **2**–**5**

For a better understanding of this section, for compounds **2**–**5**, only the ring that supports the α, β-unsaturated system was selected to discuss the charges, in e- (Table 8), due to the substitution being carried out at C3. Additionally, the sulfhydryl and primary amine groups were also considered.

Charge analysis is a powerful tool to investigate the intramolecular charge transfer that occurs between atoms in a molecule, to identify atoms or groups of atoms that play the role of electron donor or acceptor [41]. In this sense, the most negative values were observed, in general, at oxygen atoms (from −0.527 to −0.563 e-) and nitrogen atoms (−0.827 e- for **3**, and −0.771 e- for **5**). On the other hand, the most deficient charge, after carbon atoms of carbonyl groups, is the hydrogen atoms. For **2**, the hydrogen supported on the sulfur atom has a value of 0.110 e-. For **3**, the hydrogen atoms supported in the amino group display a charge of 0.351 and 0.345 e-, noting that the hydrogen with the lesser value establishes the hydrogen bond with the O=C4 group. For **4**, the hydrogen supported in the sulfur atom labeled as S19 has a value of 0.120 e-. For **5**, the hydrogen atoms supported in the amino group display a charge of 0.367 e- for both.

Consequently, the hydrogen atom of the substituents formed hydrogen bonds, confirming the interaction with the amino acid residues of PARP-1, highlighting the hydrogen bridges between Gly202 and the carbonyl group and the Tyr246 and sulfur moiety in **2**, which are key to inhibiting PARP-1 together with the catalytic triad [36]. Additionally, the compounds appear to have the right size and volume to bond to the catalytic site.

Regarding the structure–activity relationship, all molecules maintain interaction with amino acid residue Tyr246, which is key for the inhibitory activity; it is also important to mention that interaction with His201 and Tyr235 that are part of the catalytic triad is also maintained [36], but it is important to note that all molecules except **2** lack interaction with Gly202 residue.

Consequently, **2** and **3** display better inhibitory activity against PARP-1, because of the hydrogen bonds that are important to the biological activity [42]. In this sense, the second best molecule with moderate inhibitory activity (14.38 µM) was **3**, which showed a configuration like *cis* in both optimization and docking studies; thus, this feature favors the interaction with the amino acid residue Glu327, but not with Gly202, which is important to inhibitory activity [36]. In the case of **2**, which exhibits moderate inhibitory activity (13.76 µM), it undergoes a conformational change. The optimization study showed a configuration like *cis*, and for the docking study, an extended conformation was displayed. In this sense, it only showed interaction with Tyr235 residue as part of the catalytic triad, but displayed interaction with Gly202 and Tyr246, which, as mentioned above, is important to mimic the relevant role of stacking interactions in driving effective binding to the PARP-1 catalytic domain and establish the hydrogen bond donor and acceptor [36]. Additionally, for these two compounds, the substituents were maintained under the menadione moiety as the plane of reference (Figure 5).

Lee et al. [42] reported that several anthraquinone derivatives displayed inhibitory activity against PARP-1. It is highlighted that the compound NSC747854, a heteroannelated anthraquinone derivative featuring a 3-(dimethylamino)propylthio moiety, exhibits a dose-dependent manner at three different concentrations, with the maximum inhibitory activity observed at 10 µM. Also, Baptista [36] reported an anthraquinone derivative with a 2-aminoethylthio moiety, with an inhibitory IC_50_ >10 µ, bonding to amino acid residues Tyr246, Tyr325, and Glu327, part of the catalytic triad.

In another study, Martínez et al. [22] reported the production of two new sulfur derivatives of the quinone perezone, including a 4-fluoro-2-(hydroxymethyl)phenylthiol or thiophenyl acetic acid moieties displaying an IC_50_ of 0.3 µM, highlighting that these compounds show interaction with amino acid residues of the catalytic triad, Tyr235 and His201, and with the key amino acid residues, Tyr246 and Ser243 or Gly202, respectively. Also, Hernández-Rodríguez et al. [19] reported that the quinone perezone, without substituents, showed a low inhibitory activity with an IC_50_ of 181 µM.

With regard to **4**, the aromatic ring was observed on the upper side of the plane of reference menadione moiety (Figure 5) in the optimized study, but in the docking, it was positioned under the plane of reference, maintaining the aromatic ring perpendicular. This position probably avoids interaction with the amino acid residue Gly202, provoking a low inhibitory effect against PARP-1 (108.16 µM). For **5**, in both optimization and docking studies, the aromatic ring was maintained on the upper side of the plane of reference, but in docking, the aromatic ring undergoes a conformational change from perpendicular to angular, provoking interactions with two amino acids that are not essential for the inhibitory activity, Gly233 and Arg217, explaining their low inhibitory activity (216.80 µM). In these compounds, the addition of a third aromatic ring, a space linker, does not favor the π-π interactions; in this case, the steric hindrance probably avoids the proximity with the key amino acid, Gly202.

Consequently, the compounds with sulfur derivatives may improve inhibitory activity against PARP-1.

## 3. Materials and Methods

### 3.1. Synthesis of Compounds ***2***–***5***

#### 3.1.1. General

The menadione, 1,2-ethanedithiol, benzene-1,4-dithiol, cysteamine, and 4-aminobenzenethiol were purchased from Sigma Aldrich Chemistry (St. Louis, MO, USA) and used as received. The solvents *n*-hexane, ethyl acetate, and absolute methanol were technical grade and were used without further purification from Materiales y Abastos Especializados S.A. de C.V. (Zapopan, Jalisco, Mexico). The reactions were monitored using thin-layer chromatography (TLC) performed on pre-coated Merck silica gel (0.25 mm) 60F254 aluminum sheets using 90:10 of *n*-hexane–EtOAc as eluent; the purification of target molecules was carried out by preparative chromatography using the same established eluent for TLC, on pre-coated Merck silica gel 60F 254 glass sheets (Merck-Millipore, Darmstadt, Germany). The visualization was achieved using a 254 nm UV lamp (UVLS-24, Upland, CA, USA).

Melting points, uncorrected, were measured using a Fisher Scientific apparatus, Scientific Serial No. 810N0220 (Cole-Parmer, Vernon Hills, IL, USA). The mass spectrometric identifications were performed by DART^+^ and HRMS-DART^+^ (19.8 eV) using a JEOL JMS-T100LC (Direct Analysis in Real Time) spectrometer (JEOL, Tokyo, Japan). The measurements were performed using the DART^+^ experiment with PEG (polyethylene glycol) 400 as an internal reference at 6000 resolutions and triplet helium as the carrier gas at 350 °C. In the first orifice, the temperature and voltage were 120 °C and 15 V, respectively, and the voltage in the second orifice was 5 V. Elemental composition was calculated within a mass range of ±10 ppm from the measured mass. The corresponding NMR determinations were carried out using a Bruker Advance III spectrometer (San Francisco, CA, USA) at 300 MHz and 75 MHz for hydrogen and carbon nuclei, respectively, with solvents CDCl_3_ and DMSO-*d*_6_, and TMS as an internal reference. The multiplicities are reported as singlet (s), broad singlet (bs), doublet (d), triplet (t), double of doublet (dd), and multiple (m). The corresponding *δ*-chemical shifts are given in ppm, and the coupled constant (*J*) is given in Hertz (Hz).

#### 3.1.2. Synthesis of **2**–**5**

In an appropriate bottom flask vessel, 0.9873 mmol (170 mg) of menadione (**1**), 0.9980 mmol (94 mg) of 1,2-ethanedithiol, 0.9843 mmol (140 mg) of benzene-1,4-dithiol, 1.0644 mmol (80 mg) of cysteamine, or 0.9985 mmol (125 mg) of 4-aminobenzenethiol, and absolute methanol (10 mL) were mixed. Then the mixtures were treated under reflux for 16 h. The reaction progress was monitored using TLC (*n*-hexane/ethyl acetate, 90:10). The corresponding products were purified by preparative chromatography using the same mobile phase as the TLC and were obtained as red oils (**2**, **4**) and red solids (**3**, **5**) (Scheme 1).

#### 3.1.3. Spectroscopic Characterization of **2**–**5**

*2-((2-Mercaptoethyl)thio)-3-methylnaphthalene-1,4-dione* (**2**), red oil; yield 37% of isolated pure product (97.73 mg), R_f_ = 0.18; ^1^H NMR (DMSO-*d*_6_) *δ* (ppm): 7.96–7.91 (m, 2H, *J* = 8 Hz, H9 and H6), 7.76–7.75 (dd, 2H, *J* = 4 Hz, H8 and H7), 2.95 (t, 2H, H14), 2.22 (t, 2H, H13), 2.18 (s,1H, H15), 2.16 (s, 3H, H11); ^13^C NMR (DMSO-*d*_6_) *δ* (ppm): 181.76 (C1), 180.87 (C4), 147.62 (C3), 145.05 (C2), 134.29 (C7), 134.23 (C5), 132.64 (C8), 131.77 (C10), 126.36 (C6), 126.29 (C9), 67.41 (C13), 38.54 (C14), 15.54 (C11); MS-DART^+^ (19.8 eV) *m*/*z* (% ra) [assignment]: 265 (100) [M + 1]^+^, 232 (15) [M − 32]^+•^, 231 (88) [M − 33]^+^, 205 (43) [M − 59]^+^; HRMS-DART^+^ (19.8 eV): elemental composition C_13_H_13_O_2_S_2_ molecular ion protonated [M + 1]^+^ in agreement with an exact value of 265.03570 Daltons and a precise value of 265.03528 Daltons, with an error of −1.56 ppm, and complementarily, the provided unsaturation data of 9.5.
*2-((2-Aminoethyl)thio)-3-methylnaphthalene-1,4-dione* (**3**), purple amorphous solid; mp >300 °C; yield 46% of isolated pure product (112.28 mg), R_f_ = 0.21; ^1^H NMR (CDCl_3_) *δ* (ppm): 8.02–7.93 (m, 2H, *J* = 8 Hz, H9 and H6), 7.65–7.63 (dd, 2H, *J* = 8 Hz, H7 and H8), 3.66 (t, 2H, H14), 3.46 (t, 2H, H13), 2.50 (s, 2H, H15), 2.11 (s, 3H, H11); ^13^C NMR (CDCl_3_) *δ* (ppm): 185.52 (C1), 184.97 (C4), 148.15 (C3), 135.63 (C2), 134.53 (C7), 134.34 (C5), 133.61 (C8), 133.56 (C10), 127.41 (C6), 126.77 (C9), 61.84 (C13), 61.34 (C14); 14.65 (C11); MS-DART^+^ (19.8 eV) *m*/*z* (% ra) [assignment]: 247 M**^+•^** not observed, 230 (100) [M − 17]**^+^**; HRMS-DART^+^ (19.8 eV): elemental composition of C_13_H_12_NOS^+^ for molecular ion protonated [M + 1]**^+^** with loss of water molecule by cross-oxidation and cyclization via dehydration [21], in agreement with an exact value of 230.06396 Daltons and a precise value of 230.06329 Daltons, with an error of −2.89 ppm, and complementarily, the provided unsaturation data of 8.5.
*2-((4-Mercaptophenyl)thio)-3-methylnaphthalene-1,4-dione* (**4**), red oil; yield 35% of isolated pure product (109.95 mg), R_f_ = 0.13; ^1^H NMR (DMSO-*d*_6_) *δ* (ppm): 7.64–7.62 (m, 2H, *J* = 8 Hz, H6 and H9), 7.56–7.52 (dd, 1H, *J* = 4 Hz, H7 and H8), 7.41–7.40 (d, 2H, *J* = 4 Hz, H14 and H18), 7.36–7.35 (d, 2H, *J* = 4 Hz, H15 and H17), 3.92 (s, 1H, H19), 1.98 (s, 3H, H11); ^13^C NMR (DMSO-*d*_6_) *δ* (ppm): 196.38 (C1), 193.46 (C4), 140.25 (C16), 134.88 (C2), 134.50 (C7), 134.40 (C3), 134.19 (C18), 133.99 (C14), 133.82 (C5), 131.67 (C10), 129.82 (C8), 129.76 (C6), 128.20 (C9), 126.32 (C13), 126.10 (C15), 125.46 (C17), 13.93 (C11); MS-DART^+^ (19.8 eV) *m*/*z* (% ra) [assignment]: 313 (17) [M + 1]^+^, 279 (100) [M − 33]^+^, 205 (83) [M − 107]^+^; HRMS-DART^+^ (19.8 eV): elemental composition of C_17_H_13_O_2_S_2_ for molecular ion protonated [M + 1]^+^ in agreement with an exact value of 313.0570 Daltons and a precise value of 313.03528 Daltons, with an error of −1.33 ppm, and complementarily, the provided unsaturation data of 13.5.
*2-((4-Aminophenyl)thio)-3-methylnaphthalene-1,4-dione* (**5**), red amorphous solid; mp 143–145 °C; yield 35% of isolated pure product (101.63 mg), R_f_ = 0.25; ^1^H NMR (DMSO-*d*_6_) *δ* (ppm): 7.99–7.97 (dd, 1H, *J* = 8 Hz, H9), 7.91–7.89 (dd, 1H, *J* = 8 Hz, H6), 7.83–7.78 (m, 2H, *J* = 4 Hz, H7 and H8), 7.15–7.13 (d, 2H, *J* = 8 Hz, H14 and H18), 6.52–6.51 (d, 2H, *J* = 4 Hz, H15 and H17), 5.38 (s, 2H, H19), 2.12 (s, 3H, H11); ^13^C NMR (DMSO-*d*_6_) *δ* (ppm): 182.42 (C1), 180.59 (C4), 149.01 (C16), 146.20 (C2), 145.65 (C7), 134.29 (C3), 133.94 (C18), 133.74 (C14), 133.63 (C5), 131.99 (C8), 131.65 (C10), 126.19 (C6), 126.04 (C9), 120.71 (C13), 114.38 (C15), 114.12 (C17), 14.99 (C11); MS-DART^+^ (19.8 eV) *m*/*z* (% ra) [assignment]: 296 (100) [M + 1]^+^, 248 (63) [M − 47]^+^; HRMS-DART^+^ (19.8 eV): elemental composition C_17_H_14_NO_2_S for molecular ion protonated [M + 1]**^+^** in agreement with an exact value of 296.07452 Daltons and a precise value of 296.07532 Daltons, with an error of 2.69 ppm, and complementarily, the provided unsaturation data of 12.5.

### 3.2. Biological Activity Prediction

Biological activity prediction was developed employing chemoinformatic online tools, including SwissADME (version 2017) [27], Osiris Property Explorer (version 2001) [28], and ProTox 3.0 (version 2024) [29], from which physicochemical properties such as molecular weight, MLogP, hydrogen donor and acceptor, and topological polar surface area were acquired, as an approach to Lipinski’s rule of five.

### 3.3. Docking Studies

**Figure 8:**
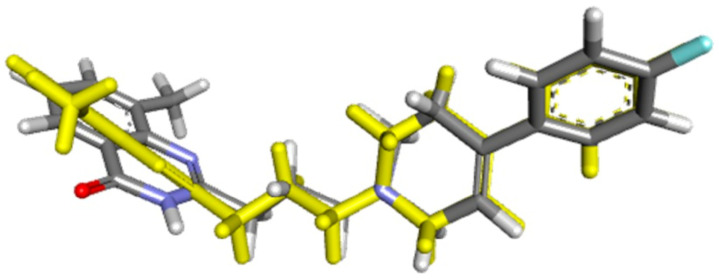
RMSD for the co-crystallized ligand FR257517 (yellow) concerning the reference ligand at the crystal structures (gray) for illustrating a good docking solution (RMSD ≤ 2.0 Å) as PARP-1 docking validation.

Docking studies were performed to determine at an atomic level the probable interaction of the target molecules with PARP-1 (PDB ID: 1UK0). Autodock 4.2 software was used for docking studies (http://autodock.scripps.edu, accessed on 15 May 2025) due to this algorithm maintaining a rigid macromolecule, allowing ligand flexibility [43,44]. This program has been widely used because it displays good correlations between free energy and binding pose values from docking simulations and corresponding experimental data [35,45]. Three-dimensional structures of compounds **1**–**5** were generated as described below. In addition, to validate the docking protocol, the known inhibitor (olaparib) for the studied protein was included in the docking study. A GRID-based procedure was utilized to prepare the structural inputs and define all the binding sites. A rectangular lattice (70 × 70 × 70 Å) with points separated by 0.375 Å was centered on the active site of PARP-1. The docking simulation was conducted using a hybrid Lamarckian genetic algorithm with an initial population of 100 randomly placed individuals and a maximum of 1.0 × 10^7^ energy evaluations. All other parameters were maintained at their default settings. Conformations with the lowest free energy binding (ΔG) and the highest frequency were selected employing AutoDock tools. The images were created by using Discovery Studio 2024 Client (https://biovia-discovery-studio-2024-client.software.informer.com/, accessed on 9 March 2026). Before the molecular coupling assay, validation of the docking protocol was performed by re-docking the co-crystallized ligand FR257517 from the original 3D structure, using the same procedure as for the docked compounds (Figure 8, RMSD 1.1054 Å).

### 3.4. PARP-1 Inhibition Assay

To corroborate the PARP-1 inhibition by **2**–**5**, PARP-1 activity was determined in their absence and presence, employing a colorimetric assay kit (BPS Bioscience Inc., San Diego, CA, USA, Catalog number 80580), according to manufacturer’s instructions, employing olaparib (BPS Bioscience Inc., San Diego, CA, USA, Catalog number 27003) as a reference compound at a crescent concentration (0.0001, 0.0011, 0.0115, 0.1151, 1.1507, and 11.5075 µM, *n* = 3); 6.6 ± 2.0 mg of the reference compound and the tested compounds was weighed to achieve a standard solution of 32.57, 19.43, 18.33, and 16.95 µM concentration (*n* = 3). Serial dilutions were made to achieve µM concentrations near the reference compound. Absorbances were obtained at 450 nm using a UV/Vis spectrophotometer Multiskan FC ELISA (ThermoFisher Scientific, Waltham, MA, USA). The absorbance data were entered into GraphPad Prism 8.0.1 software (2026 GraphPad Software by Dotmatics) using the Dose–Response–Inhibition option to calculate the IC50 and statistical parameters from the data.

### 3.5. Quantum Chemical Calculations

A conformational analysis at the molecular mechanical level, using Spartan06 (Version 06, Wavefunction, Inc., Irvine, CA, USA) [46], was performed. The Becke’s three-parameter hybrid density functional B3LYP [47,48] of the DFT level of theory [49,50,51] with the 6-311G(d,p) basis set [52,53,54] in the gas phase using Gaussian 16 (Version 2016.03, Gaussian, Inc., 340 Quinipiac St., Bldg. 40, Wallingford CT 06492, UK) [55] software, and visualized by the GaussView [56] application, was used to calculate geometry optimization and electronic properties, molecular electrostatic potential (MEP) [57], natural population analysis (NPA) [58] by the natural bond orbital (NBO) program [54] implemented in Gaussian 16 software, frontier molecular orbital [59], and DFT global chemical reactivity descriptors [60,61]. The optimization was carried out using the Berny analytical gradient optimization methods [62]. In all cases, default convergence criteria were used. Optimized geometries were also used to reproduce ^1^H and ^13^C NMR chemical shifts obtained using the Gauge-Independent Atomic Orbital (GIAO) approach [63,64] with global scaling factors of 31.7553 and 191.8156 for ^1^H and ^13^C, respectively, concerning TMS in the gas phase.

## 4. Conclusions

Four new menadione derivatives were produced through a Michael addition protocol. Therefore, all compounds were analyzed at the quantum level (geometrical, electronic, spectroscopic, and reactivity properties). The obtained molecules were unequivocally characterized by common spectroscopic data, and their comparison with theoretical ^1^H and ^13^C NMR spectra showed a good correlation, considering the solvent effect. To know the sites that could interact with the amino acid residues of the PARP-1 protein, theoretical calculations employing DFT using the B3LYP/6-311G(d,p) method were performed. In this sense, the reactivity properties studies and HOMO-LUMO of all molecules showed that **4** and **5** were the most reactive, but the nucleophilicity of **3** and **2** improved the affinity to PARP-1. The docking studies of the compounds indicated that molecules **2** and **3** have the worst free binding energy, −7.97 and −9.35 kcal/mol, in comparison with **4**, **5**, and olaparib, but the best PARP-1 inhibitory activity, 13.76 µM for **2** and 14.38 µM for **3**. Also, it was evidenced that the amino acid residues Gly202 and Tyr246 are key to the inhibitory activity, together with the catalytic triad.

According to their IC_50_, compounds that support an aromatic ring as a space linker diminished the activity against PARP-1. On the other hand, compounds that support an alkyl group with a substituent that forms a hydrogen bond (sulfhydryl or amino group) promote better inhibitory activity against PARP-1. In this sense, **2** and **3** displayed hydrogen bonds with amino acid residues Tyr246, Gly202, and Glu327, respectively, that are key for the inhibitory activity. Consequently, compound **2** was the only compound that showed interaction between the sulfur atom and the amino acid residue Tyr246, establishing the stacking interaction and promoting the hydrogen bond with amino acid residue Gly202; thus, this compound with a sulfur atom in its skeleton should be considered as a potential antineoplastic agent, due to maintaining similar interactions to olaparib and because the biological prediction study does not display mutagenic, tumorigenic, or irritating risk and shows good gastrointestinal absorption.

## Figures and Tables

**Table 1:** ^1^H and ^13^C NMR theoretical and experimental chemical shifts (*δ*, ppm) for **2**–**5**.

Hydrogen ^1^	2	3	4	5
	*δ* (ppm) Calculated/Calculated Solvent ^2^/Experimental			
6	8.07/8.17/7.96–7.91	8.10/8.17/8.02–7.93	7.81/8.11/7.64–7.62	7.80/7.90/7.91–7.89
7	7.48/7.89/7.76–7.75	7.51/7.89/7.65–7.63	7.41/8.02/7.56–7.52	7.38/7.82/7.83–7.78
8	7.51/7.91/7.76–7.75	7.54/7.91/7.65–7.63	7.46/8.06/7.56–7.52	7.43/7.87/7.83–7.78
9	8.10/8.17/7.96–7.91	8.10/8.17/8.02–7.93	8.07/8.36/7.64–7.62	8.06/8.16/7.99–7.97
11	2.12/2.19/2.16	2.16/2.18/2.11	2.24/2.51/1.98	2.24/2.32/2.12
13	3.23/3.28/2.22	3.00/3.22/3.46	-	-
14	2.54/2.83/2.95	2.70/2.88/3.66	6.92/7.25/7.41–7.40	6.97/7.12/7.15–7.13
15	1.47/2.07/2.18	1.11/0.69/2.50	6.71/7.21/7.36–7.35	6.17/6.57/6.52–6.51
16	-	-	-	-
17	-	-	6.84/7.47/7.36–7.35	6.40/6.75/6.52–6.51
18	-	-	7.65/7.79/7.41–7.40	7.82/7.68/7.15–7.13
19	-	-	3.50/4.49/3.92	2.77/3.67/5.38
**Carbon** ^1^				
1	191.89/188.53/181.76	192.29/188.26/185.52	193.95/190.31/196.38	193.86/190.03/182.42
2	158.64/155.34/145.05	162.29/154.85/135.63	160.64/159.38/134.88	159.05/156.24/146.20
3	169.15/163.42/147.62	166.74/163.94/148.15	166.60/158.03/134.40	167.81/160.15/134.29
4	194.26/189.14/180.87	192.89/189.01/184.97	191.92/187.44/193.46	192.34/188.02/180.59
5	145.02/139.32/134.23	145.36/139.43/134.34	145.19/139.17/133.82	145.51/139.50/133.63
6	140.26/134.62/126.36	140.53/134.57/127.41	140.40/134.67/129.76	140.09/134.40/126.19
7	145.40/141.93/134.29	145.60/141.84/134.53	145.80/142.35/134.50	145.33/142.04/145.65
8	146.39/142.43/132.64	146.57/142.35/133.61	145.89/142.34/129.82	145.49/142.05/131.99
9	140.41/134.35/126.29	140.48/134.31/126.77	140.06/134.31/128.20	139.91/134.07/126.04
10	144.35/138.25/131.77	144.48/138.25/133.56	144.11/138.14/131.67	144.27/138.20/131.65
11	23.52/17.36/15.54	24.55/17.18/14.65	24.47/18.96/13.93	24.02/18.04/14.99
13	57.70/52.21/67.41	57.12/55.10/61.84	148.13/145.08/126.32	137.76/131.71/120.71
14	43.60/38.03/38.54	55.08/50.57/61.34	147.45/139.58/133.99	150.16/142.71/133.74
15	-	-	137.10/132.99/126.10	124.17/120.16/114.38
16	-	-	153.99/145.74/140.25	162.53/158.69/149.01
17	-	-	139.33/135.31/125.46	124.88/119.89/114.12
18	-	-	150.38/139.14/134.19	153.74/143.40/133.94

^1^ The numeration according to Figure 1. ^2^ The chemical shift values consider the solvent effect (DMSO).

**Table 2:** Lipinski rule of 5 predicted properties for **1**–**5**.

Properties	Compounds				
	1	2	3	4	5
Molecular weight (g/mol)	172.18	264.36	247.31	312.41	295.36
MLogP ^1^/WLogP ^2^	1.20/2.01	1.76/3.0	0.88/2.03	3.07/4.42	2.19/3.72
nON	2	2	3	2	2
nOHNH	0	0	1	0	1
TPSA (Å)	34.14	98.24	85.46	98.24	85.46

^1^ MLogP [31] was employed as an atomistic method for analysis and suggested by Lipinski [30]. ^2^ WLogP [32] was suggested for the boiled egg diagram representation.

**Table 3:** Pharmacokinetic ADME-Tox prediction for **1**–**5**.

Properties ^1^	1	2	3	4	5
Gastrointestinal absorption	High	High	High	High	High
Blood–brain barrier permeant	Yes	No	No	No	No
P-gp substrate	No	No	No	No	No
CYP1A2 inhibitor	Yes	Yes	Yes	Yes	Yes
CYP2C19 inhibitor	No	Yes	Yes	Yes	Yes
CYP2C9 inhibitor	No	Yes	No	Yes	Yes
CYP2D6 inhibitor	No	No	No	No	No
CYP3A4 inhibitor	No	No	No	Yes	Yes
Mutagenic risk	Yes	No	No	No	No
Tumorigenic risk	Yes	No	No	No	No
Irritating risk	No	No	No	No	No
Reproductive effect risk	Yes	No	No	No	No
Drug score	0.1	0.38	0.55	0.27	0.48
Predicted toxicity class	4	3	3	4	4

^1^ Pharmacokinetic property obtained by the SwissADME predictor [27]. Estimated drug score and toxicity by Osiris Property Explorer, preferred when closest to 1.0 [28]. Predicted toxicity class results from ProTox 3.0 [29].

**Table 4:** Inhibitory concentration (IC_50_) values in µM for **1**–**5** and olaparib, evaluated against the PARP-1 enzyme (mean ± S.D. of three values).

Compound	IC_50_ (µM) Against the PARP-1 Enzyme
**1**	25 ^1^
**2**	13.76 ± 22.51
**3**	14.38 ± 32.01
**4**	108.16 ± 71.24
**5**	216.80 ± 124.09
Olaparib	70.09 ± 17.77

^1^ Value from Reference [37].

**Table 5:** Bond length in Å calculated for menadione thioderivatives **2**–**5**.

Bond	Experimental Data for 1		Theoretical Data			
	Ref. [38]	Ref. [39] ^1^	2	3	4	5
C1=O	1.229	1.202	1.221	1.221	1.221	1.221
C1-C2	1.453	1.515	1.493	1.495	1.496	1.498
C2-C11	1.491	1.467	1.500	1.500	1.500	1.501
C2-C3	1.366	1.272	1.363	1.364	1.360	1.361
C3-C4	1.484	1.581	1.499	1.501	1.502	1.502
C3-H	1.081	0.930	-	-	-	-
C4=O	1.231	1.219	1.219	1.220	1.215	1.216
C4-C5	1.467	1.442	1.490	1.490	1.491	1.492
C5-C10	1.401	1.425	1.400	1.400	1.400	1.400
C5-C6	1.392	1.354	1.397	1.397	1.397	1.397
C6-C7	1.398	1.461	1.390	1.390	1.390	1.390
C7-C8	1.387	1.342	1.397	1.397	1.397	1.397
C8-C9	1.389	1.447	1.391	1.391	1.390	1.390
C9-C10	1.398	1.374	1.396	1.396	1.397	1.397
C10-C1	1.479	1.470	1.489	1.488	1.489	1.488
C3-S12	-	-	1.775	1.776	1.783	1.784
S12-C13	-	-	1.852	1.855	1.797	1.796
C13-C14	-	-	1.525	1.539	1.397	1.396
C14-S15	-	-	1.841	-	-	-
S15-H	-	-	1.350	-	-	-
C14-N15	-	-	-	1.456	-	-
N15-H	-	-	-	1.016	-	-
N15-H	-	-	-	1.016	-	-
C14-C15	-	-	-	-	1.390	1.387
C15-C16	-	-	-	-	1.398	1.405
C16-S19	-	-	-	-	1.786	-
S19-H	-	-	1.347	-	-	-
C16-N19	-	-	-	-	-	1.390
N19-H	-	-	1.009	-	-	-
N19-H	-	-	1.009	-	-	-
C16-C17	-	-	-	-	1.399	1.404
C17-C18	-	-	-	-	1.390	1.388
C18-C13	-	-	-	-	1.396	1.397

^1^ The single crystal was obtained in MeOH at 300 K.

**Table 7:** Global reactivity descriptors of neutral, positive, and negative energies in Hartrees, and energy gap (ΔE_Gap_), ionization potential (IP), electron affinity (EA), electronegativity (χ), chemical potential (µ), electrophilicity index (ω), and chemical hardness (η), in eV, for **2**–**5**.

Parameter	Compounds			
	2	3	4	5
ΔE_Gap_^1^	3.158	3.088	2.891	2.680
E_neutral_	−1449.652340	−1106.803678	−1602.106851	−1259.268162
E_positive_	−1449.356762	−1106.503052	−1601.825694	−1258.996961
E_negative_	−1449.712641	−1106.866601	−1602.168135	−1259.323342
IP ^2^	8.04	8.18	7.65	7.38
EA ^3^	1.64	1.71	1.66	1.50
χ ^4^	4.84	4.95	4.65	4.44
µ ^5^	−4.84	−4.95	−4.65	−4.44
ω ^6^	3.66	3.78	3.63	3.35
η ^7^	3.20	3.23	2.99	2.93

^1^ Energy gap. ^2^ Ionization potential. ^3^ Electron affinity. ^4^ Electronegativity. ^5^ Chemical potential. ^6^ Electrophilicity index. ^7^ Chemical hardness.

**Table 8:** Charges, in e-, of compounds **2**–**5**.

Atom ^1^	2	3	4	5
C1	0.523	0.523	0.526	0.525
C2	−0.049	−0.042	−0.043	−0.049
C3	−0.208	−0.208	−0.204	−0.200
C4	0.524	0.529	0.523	0.523
O=C1	−0.541	−0.538	−0.538	−0.542
O=C4	−0.548	−0.563	−0.527	−0.528
S12	0.269	0.235	0.289	0.283
S15	−0.034	-	-	-
H-S15	0.110	-	-	-
N15	-	−0.827	-	-
H_1_-N15	-	0.351	-	-
H_2_-N15	-	0.345	-	-
S19	-	-	0.046	-
H-S19	-	-	0.120	-
N19	-	-	-	−0.771
H_1_-N19	-	-	-	0.367
H_2_-N19	-	-	-	0.367

^1^ The numeration is according to Figure 1.

**Table 6:** Bond angles in degrees for menadione thioderivatives **2**–**5**.

Bond	Experimental Data	Theoretical Data			
	Ref. [38]/[39] ^1^	2	3	4	5
O-C1-C2	119.8/119.089	120.713	120.773	121.008	120.842
C1-C2-C3	120.4/120.978	120.712	120.801	120.389	120.535
C1-C2-C11	119.6/116.345	115.876	115.600	115.420	115.461
C11-C2-C3	120.0/122.677	123.412	123.600	124.191	124.004
C2-C3-H	119.3/118.842	-	-	-	-
C2-C3-S12	-	118.857	118.926	121.647	120.044
C2-C3-C4	121.1/122.207	121.626	121.393	121.870	121.613
H-C3-C4	119.6/118.951	-	-	-	-
C3-S12-C13	-	105.105	106.515	120.636	120.319
S12-C3-C4	-	119.019	119.192	117.579	117.904
C3-C4-O	119.7/117.662	121.606	121.857	121.783	121.647

^1^ The single crystal was obtained in MeOH at 300 K.

## Data Availability

The data reported in this study are available upon request to atlanta126@gmail.com, jomtz@comunidad.unam.mx, or mirruv@yahoo.com.mx.

## Supplementary Materials

The following supporting information can be downloaded at: https://www.mdpi.com/article/10.3390/molecules31060958/s1, Figure S1. ^1^H NMR (DMSO-*d*_6_/TMS, 300 MHz) of **2**. Figure S2. ^13^C NMR (DMSO-*d*_6_/TMS, 75 MHz) of **2**. Figure S3. MS-DART^+^ (19.8 eV) of **2**. Figure S4. HRMS-DART^+^ (19.8 eV) of **2**. Figure S5. ^1^H NMR (DMSO-*d*_6_/TMS, 300 MHz) of **3**. Figure S6. ^13^C NMR (DMSO-*d*_6_/TMS, 75 MHz) of **3**. Figure S7. MS-DART^+^ (19.8 eV) of **3**. Figure S8. HRMS-DART^+^ (19.8 eV) of **3**. Figure S9. ^1^H NMR (DMSO-*d*_6_/TMS, 300 MHz) of **4**. Figure S10. ^13^C NMR (DMSO-*d*_6_/TMS, 75 MHz) of **4**. Figure S11. MS-DART^+^ (19.8 eV) of **4**. Figure S12. HRMS-DART^+^ (19.8 eV) of **4**. Figure S13. ^1^H NMR (DMSO-*d*_6_/TMS, 300 MHz) of **5**. Figure S14. ^13^C NMR (DMSO-*d*_6_/TMS, 75 MHz) of **5**. Figure S15. MS-DART^+^ (19.8 eV) of **5**. Figure S16. HRMS-DART^+^ (19.8 eV) of **5**.

## References

[1] Siegel R.L., Miller K.D., Fuchs H.E., Jemal A. (2021). Cancer statistics, 2021. CA Cancer J. Clin..

[2] Rudolph J., Jung K., Luger K. (2022). Inhibitors of PARP: Number crunching and structure gazing. Proc. Natl. Acad. Sci. USA.

[3] Jackson S.P., Bartek J. (2009). The DNA-damage response in human biology and disease. Nature.

[4] Shanmugam N., Chatterjee S., Cisneros G.A. (2025). Impact of a cancer-associated mutation on poly(ADP-ribose) polymerase1 inhibition. J. Phys. Chem. B.

[5] Passeri D., Camaioni E., Liscio P., Sabbatini P., Ferri M., Carotti A., Giachè N., Pellicciari R., Gioiello A., Macchiarulo A. (2016). Concepts and molecular aspects in the polypharmacology of PARP-1inhibitors. ChemMedChem.

[6] Wang L., Liang C., Li F., Guan D., Wu X., Fu X., Lu A., Zhang G. (2017). PARP1 in carcinomas and PARP1 inhibitors as antineoplastic drugs. Int. J. Mol. Sci..

[7] Oliver F.J., de la Rubia G., Rolli V., Ruiz-Ruiz M.C., de Murcia G., Ménissier-de Murcia J. (1998). Importance of poly(ADP-ribose) polymerase and its cleavage in apoptosis: Lesson from an uncleavable mutant. J. Biol. Chem..

[8] Alemasova E.E., Lavrik O.I. (2019). Poly(ADP-ribosyl)ation by PARP1: Reaction mechanism and regulatory proteins. Nucleic Acids Res..

[9] Hassan G.S., Britain H.G. (2013). Menadione. Profiles of Drug Substances, Excipients and Related Methodology.

[10] Acharya B.R., Choudhury D., Das A., Chakrabarti G. (2009). Vitamin K3 disrupts the microtubule networks by binding to tubulin: A novel mechanism of its antiproliferative activity. Biochemistry.

[11] Ngo E.O., Sun T.-P., Chang J.-Y., Wang C.-C., Chi K.-H., Cheng A.-L., Nutter L.M. (1991). Menadione-induced DNA damage in a tumor cell line. Biochem. Pharmacol..

[12] Nishikawa Y., Carr B.I., Wang M., Kar S., Finn F., Dowd P., Zheng Z.B., Kerns J., Naganathan S. (1995). Growth inhibition of hepatoma cell induced by vitamin k and its analogs. J. Biol. Chem..

[13] Wellington K.W., Hlatshwayo V., Kolesnikova N.I., Taru Saha S., Kaur M., Motadi L.R. (2020). Anticancer activities of vitamin K3 analogues. Investig. New Drugs.

[14] de Souza A.S., Ribeiro R.C.B., Costa D.C.S., Pauli F.P., Pinho D.R., de Moraes M.G., da Silva F.C., Forezi L.S.M., Ferreira V.F. (2022). Menadione: A platform and target to valuable compounds synthesis. Beilstein J. Org. Chem..

[15] Wellington K.W., Gordon G.E.R., Ndlovu L.A., Steenkamp P. (2013). Laccase-catalyzed C-S and C-C coupling for a one-pot synthesis of 1,4-naphthoquinones sulfides and 1,4-naphthoquinones sulfide dimers. ChemCatChem.

[16] Chen C., Liu Y.-Z., Shia K.-S., Tseng H.-Y. (2002). Synthesis and anticancer evaluation of vitamin K3 analogues. Bioorg. Med. Chem. Lett..

[17] Wellington K.W., Kolesnikova N.I. (2012). A laccase-catalysed one-pot synthesis of aminonaphthoquinones and their anticancer activity. Bioorg. Med. Chem..

[18] Wellington K.W., Kolesnikova N.I., Nyoka N.B.P., McGaw L.J. (2019). Investigation of the antimicrobial and anticancer activity of aminonaphthoquinones. Drug. Dev. Res..

[19] Hernández-Rodríguez M., Mendoza-Sánchez P.I., Macias Perez M.E., Rosales Cruz E., Mera Jiménez E., Nicolás Vázquez M.I., Miranda Ruvalcaba R. (2019). In vitro and computational studies showed that perezone inhibits PARP-1 and induces changes in the redox state of K562 cells. Arch. Biochem. Biophys..

[20] Hernández-Rodríguez M., Mendoza-Sánchez P.I., Martínez J., Macías Pérez M.E., Rosales Cruz E., Żołek T., Maciejewska D., Miranda Ruvalcaba R., Mera Jiménez E., Nicolás-Vázquez M.I. (2022). In vitro and Computational studies of perezone and perezone angelate as potential anti-glioblastoma multiforme agents. Molecules.

[21] Ballinas-Indilí R., Nicolás-Vázquez M.I., Martínez J., Ramírez-Apan T., Álvarez-Toledano C., Toscano A., Hernández-Rodríguez M., Mera Jiménez E., Miranda Ruvalcaba R. (2023). Synthesis, cytotoxic activity and in silico Study of novel dihydropyridine carboxylic acids derivatives. Int. J. Mol. Sci..

[22] Rubiales-Martínez A., Martínez J., Mera-Jiménez E., Pérez-Flores J., Téllez-Isaías G., Miranda Ruvalcaba R., Hernández-Rodríguez M., Mancilla Percino T., Macías Pérez M.E., Nicolás-Vázquez M.I. (2024). Design of two new sulfur derivatives of perezone: In silico study simulation targeting PARP-1 and in vitro study validation using cancer cell lines. Int. J. Mol. Sci..

[23] Martínez J., Hernández-Rodríguez M., Escobedo-González R., Nicolás-Vázquez M.I., Saavedra-Leos Z., Miranda Ruvalcaba R. (2019). Computational characterization of perezone, isoperezone and their sulfur-derivatives: Anti-inflammatory activity. ChemistrySelect.

[24] Fragoso-Medina A.J., Escobedo-González R.G., Nicolás-Vázquez M.I., Arroyo-Razo G.A., Noguez-Córdova M.O., Miranda-Ruvalcaba R. (2017). A DFT study of the geometrical, spectroscopical and reactivity properties of diindolylmethane-phenylboronic acid hybrids. Molecules.

[25] Martínez J., Hernández Rodríguez M., Miranda Ruvalcaba R., Escobedo-González R., Nicolás-Vázquez M.I. (2021). Can (*S*)-stereoisomers of perezone and its derivatives show similar activity to its (*R*)-stereoisomers? A Computational characterization and docking study. ChemistrySelect.

[26] Finley K.T., Patai S. (1974). The addition and substitution chemistry of quinones. The Chemistry of Quinoid Compounds.

[27] Daina A., Michielin O., Zoete V. (2017). SwissADME: A free web tool to evaluate pharmacokinetics, drug-likeness, and medicinal chemistry friendliness of small molecules. Sci. Rep..

[28] Sander T. OSIRIS Property Explorer. Organic Chemistry Portal, 2001. https://www.organic-chemistry.org/prog/peo.

[29] Wu F., Zhou Y., Li L., Shen X., Chen G., Wang X., Liang X., Tan M., Huang Z. (2020). Computational approaches in preclinical studies on drug discovery and development. Front. Chem..

[30] Lipinski C.A., Lombardo F., Dominy B.W., Feeney P.J. (1997). Experimental and computational approaches to estimate solubility and permeability in drug discovery and development settings. Adv. Drug Deliv. Rev..

[31] Moriguchi I., Hirono S., Liu Q., Nakagome I., Matsushita Y. (1992). Simple method of calculating octanol/water partition coefficient. Chem. Pharm. Bull..

[32] Wildman S., Crippen G. (1999). Prediction of physicochemical parameters by atomic contribution. J. Chem. Inf. Model..

[33] Daina A., Zoete V. (2016). A BOILED-Egg to predict gastrointestinal absorption and brain penetration of small molecules. ChemMedChem.

[34] Meng X.-Y., Zhang H.-X., Mezei M., Cui M. (2011). Molecular docking: A powerful approach for structure-based drug discovery. Curr. Comput. Aided Drug Des..

[35] Escobedo González R., Vargas-Requena C.L., Moyers-Montoya E., Aceves-Hernández J.M., Nicolás-Vázquez M.I., Miranda-Ruvalcaba R. (2017). *In silico* study of the pharmacologic properties and cytotoxicity pathways in cancer cells of various indolylquinones analogues of perezone. Molecules.

[36] Baptista S.J., Silva M.M.C., Moroni E., Meli M., Colombo G., Dinis T.C.P., Salvador J.A.R. (2017). Novel PARP-1 inhibitor scaffolds disclosed by a dynamic structure-based pharmacophore approach. PLoS ONE.

[37] Oztopcu-Vatan P., Sayitoglu M., Gunindi M., Inan E. (2015). Cytotoxic and apoptotic effects of menadione on rat hepatocellular carcinoma cells. Cytotechnology.

[38] Nowell H., Attfield J.P. (2004). X-ray and neutron powder diffraction studies of the crystal structure of vitamin K_3_. New J. Chem..

[39] Rane S., Ahmed K., Salunke-Gawali S., Zaware S.B., Srinivas D., Gonnade R., Bhadbhade M. (2008). Vitamin K_3_ family members–part II: Single crystal X-ray structures, temperature-induced packing polymorphism, magneto-structural correlations and probable anti-oncogenic candidature. J. Mol. Struct..

[40] Abdou A., Omran O.A., Nafady A., Nafady A., Antipin I.S. (2022). Structural, spectroscopic, FMOs, and non-linear optical properties exploration of three thiacaix(4)arenes derivatives. Arabian J. Chem..

[41] Prabhu C., Rajesh P., Dhanalakshmi E., Gnanasambandan T., Priyadharshini M. (2023). Structure conformational, molecular docking and computational investigation of methyl linoleate. Chem. Phys. Impact.

[42] Lee Y.-R., Yu D.-S., Liang Y.-C., Huang K.-F., Chou S.-J., Chen T.-C., Lee C.-C., Chen C.-L., Chiou S.-H., Huang H.-S. (2013). New approaches of PARP-1 inhibitors in human lung cancer cells and cancer stem-like cells by some selected anthraquinone-derived small molecules. PLoS ONE.

[43] Huey R., Morris G.M., Olson A.J., Goodsell D.S. (2007). A semiempirical free energy force field with charge-based desolvation. J. Comput. Chem..

[44] Morris G.M., Huey R., Lindstrom W., Sanner M.F., Belew R.K., Goodsell D.S., Olson A.J. (2009). AutoDock4 and AutoDockTools4: Automated docking with selective receptor flexibility. J. Comput. Chem..

[45] Ramírez-Durán L.A., Rosales-Hernández M.C., Hernández-Rodríguez M., Mendieta-Wejebe J.E., Trujillo-Ferrara J., Correa-Basurto J. (2013). Mapping myeloperoxidase to identify its promiscuity properties using docking and molecular dynamics simulations. Curr. Pharm. Des..

[46] Deppmeier B.J., Driessen A.J., Hehre T.S., Hehre W.J., Johnson J.A., Klunzinger P.E., Leonard J.M., Pham I., Pietro W.J., Jianguo Y. (2006). In Spartan 06 for Windows.

[47] Becke A.D. (1988). Density functional exchange-energy approximation with correct asymptotic behavior. Phys. Rev. A.

[48] Lee C., Yang W., Parr R.G. (1988). Development of the Colle-Salvetti correlation-energy formula into a functional of the electron density. Phys. Rev. B.

[49] Parr R.G., Fukui K., Pulman B. (1980). Density Functional Theory of Atoms and Molecules. Horizon of Quantum Chemistry, Proceedings of the Third International Congress of Quantum Chemistry, Kyoto, Japan, 29 October–3 November 1979.

[50] Kohn W., Becke A.D., Parr R.G. (1996). Density functional theory of electronic structure. J. Phys. Chem..

[51] Medvedev M.G., Bushmarinov I.S., Sun J., Perdew J.P., Lyssenko K.A. (2017). Density functional theory is staying from the path toward the exact functional. Science.

[52] Ditchfield R., Hehre W.J., People J.A. (1971). Self-consistent molecular-orbital methods. IX. An extended Gaussian-type basis for molecular-orbital studies of organic molecules. J. Chem. Phys..

[53] Hariharan P.C., People J.A. (1973). The influence of polarization functions on molecular orbital hydrogenation energies. Theor. Chim. Acta.

[54] Wiberg K.B. (2004). Basis set effects on calculated geometries: 6-311++G** vs. aug-cc-pVDZ. J. Comput. Chem..

[55] Frisch M.J., Trucks G.W., Schlegel H.B., Scuseria G.E., Robb M.A., Cheeseman J.R., Scalmani G., Barone V., Mennucci B., Petersson G.A. (2013). Gaussian 09, Revision E.01.

[56] Dennington R.D., Keith T.A., Millam J.M. (2016). GaussView 6.0.16 (64-Bit Windows).

[57] Du Q.S., Huang R.B., Chou K.C. (2010). Advances in visual representation of molecular potentials. Expert Opin. Drug Discov..

[58] Reed A.E., Weinstock R.B., Weinhold F. (1985). Natural populations analysis. J. Chem. Phys..

[59] Glendening E.D., Badenhoop J.K., Reed A.E., Carpenter J.E., Bohmann J.A., Morales C.M., Weinhold F. (1998). NBO, Version 3.

[60] Zhang G., Musgrave C.B. (2007). Comparison of DFT methods for molecular orbital eigenvalue calculations. J. Phys. Chem. A.

[61] Parr R.G., Donnelly R.A., Levy M., Palke W.E. (1978). Electronegativity: The density functional viewpoint. J. Chem. Phys..

[62] Schlegel H.B. (1982). Optimization of equilibrium geometries and transition structures. J. Comput. Chem..

[63] Wolinski K., Hilton J.F., Pulay P. (1990). Efficient implementation of the gauge-independent atomic orbital method for NMR chemical shift calculation. J. Am. Chem. Soc..

[64] Cheeseman J.R., Trucks G.W., Keith T.A., Frisch M.J. (1996). A comparison of models for calculating nuclear magnetic resonance shieldings tensors. J. Chem. Phys..

