# Supplementary material for: Four New Menadione Thioderivatives, Potential Antineoplastic Candidates: In Silico and PARP-1 Inhibition Studies
Source: Molecules. 2026 Mar 12;31(6):958. doi: 10.3390/molecules31060958 (PMC13028785; doi:10.3390/molecules31060958)
Supplement: Supplementary file 1 [file molecules-31-00958-s001.zip › molecules-4115519-supplementary.pdf]

Four New Menadione Thioderivatives, Potential Antineoplastic Candidates: In Silico and PARP-1 Inhibition Studies

Francisco Javier Pérez Flores [1], Luis Jaime Vázquez-López [2], Adriana Lizbeth Rivera Espejel [2], María Inés Nicolás-Vázquez [2], María Z. Saavedra-Leos [3], Alberto A. Fajardo de la Rosa [4], Samuel Álvarez-Almazán [5], Joel Martínez [2,*] and René Miranda Ruvalcaba [2,*]

* Correspondence: jomtz@comunidad.unam.mx or atlanta126@gmail.com (J.M.); mirruv@comunidad.unam.mx (R.M.R.)

CONTENT

Figure S1. ^{1}H NMR (DMSO-d_6/TMS, 300 MHz) of **2**.

Figure S2. ^{13}C NMR (DMSO-d_6/TMS, 75 MHz) of **2**.

Figure S3. MS-DART^{+} (19.8 eV) of **2**.

Figure S4. HRMS-DART^{+} (19.8 eV) of **2**.

Figure S5. ^{1}H NMR (DMSO-d_6/TMS, 300 MHz) of **3**.

Figure S6. ^{13}C NMR (DMSO-d_6/TMS, 75 MHz) of **3**.

Figure S7. MS-DART^{+} (19.8 eV) of **3**.

Figure S8. HRMS-DART^{+} (19.8 eV) of **3**.

Figure S9. ^{1}H NMR (DMSO-d_6/TMS, 300 MHz) of **4**.

Figure S10. ^{13}C NMR (DMSO-d_6/TMS, 75 MHz) of **4**.

Figure S11. MS-DART^{+} (19.8 eV) of **4**.

Figure S12. HRMS-DART^{+} (19.8 eV) of **4**.

Figure S13. ^{1}H NMR (DMSO-d_6/TMS, 300 MHz) of **5**.

Figure S14. ^{13}C NMR (DMSO-d_6/TMS, 75 MHz) of **5**.

Figure S15. MS-DART^{+} (19.8 eV) of **5**.

Figure S16. HRMS-DART^{+} (19.8 eV) of **5**.

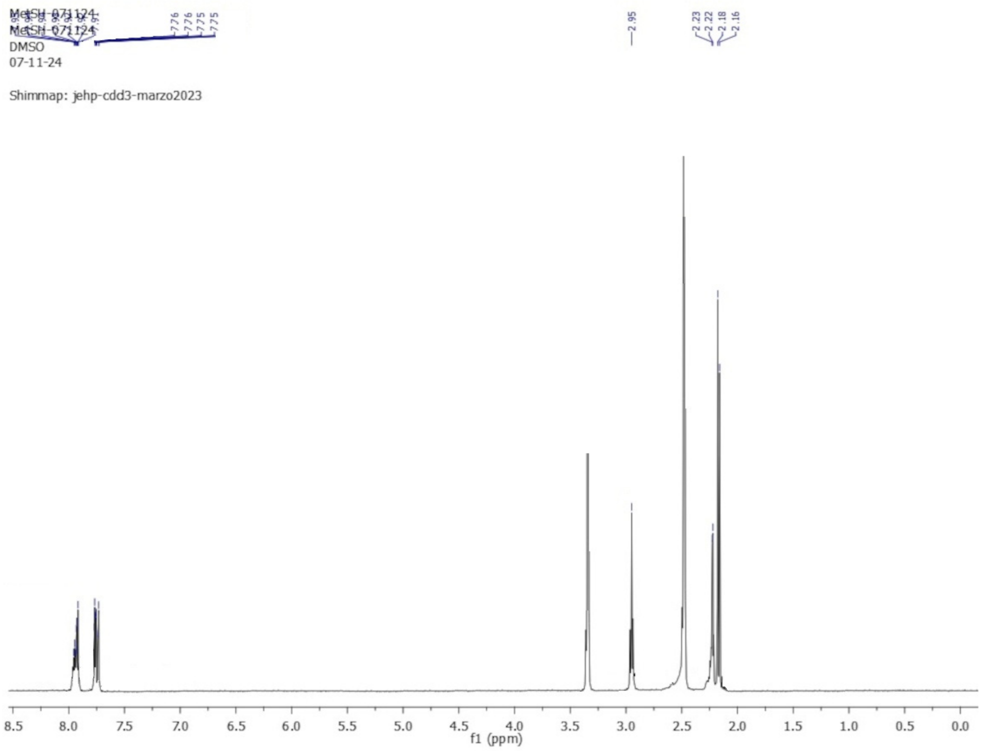

Figure S1. ^{1}H NMR (DMSO-d_6/TMS, 300 MHz) of **2**.

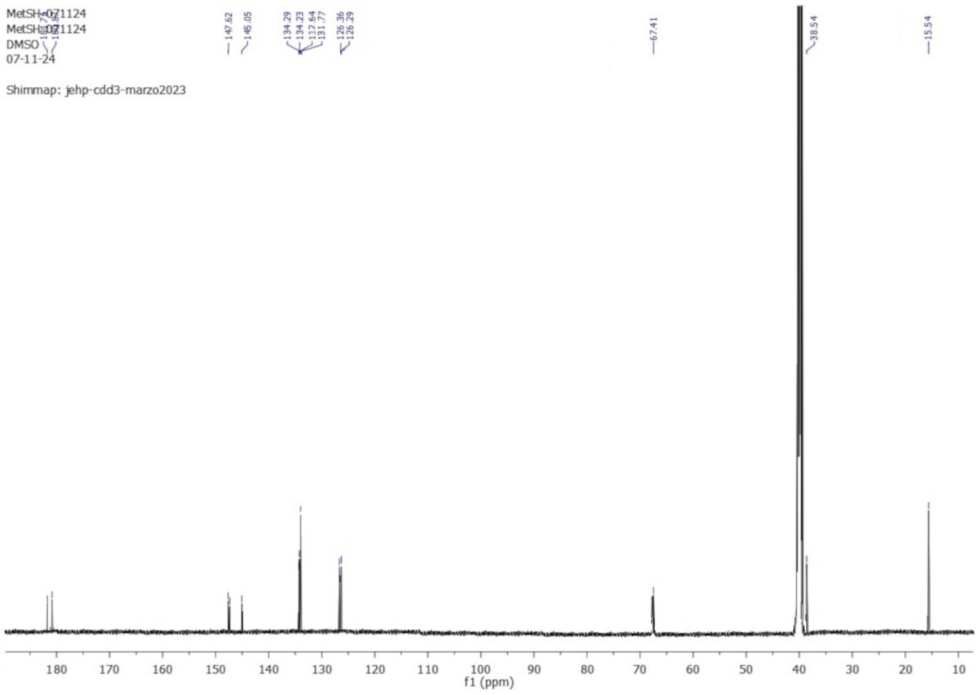

Figure S2. ^{13}C NMR (DMSO-d_6/TMS, 75 MHz) of **2**.

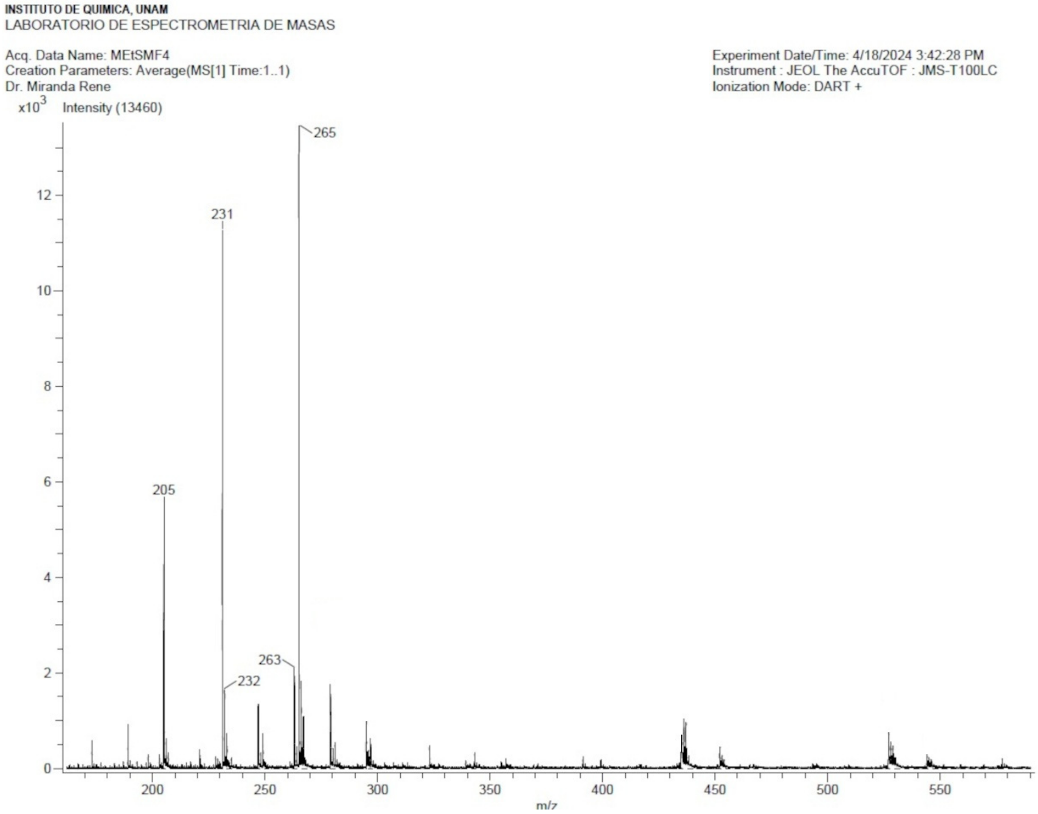

Figure S3. MS-DART$^+$ (19.8 eV) of **2**.

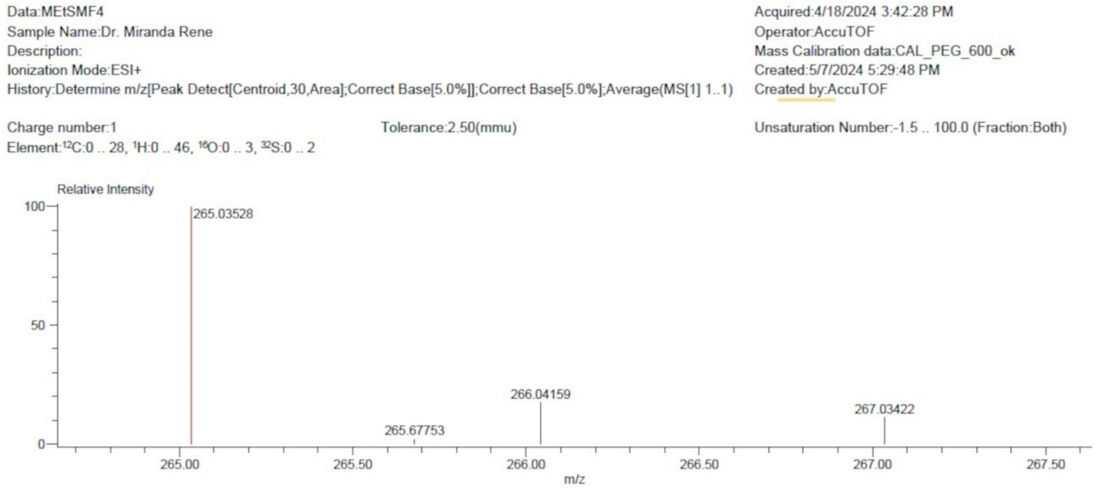

Mass	Intensity	Calc. Mass	Mass Difference (mmu)	Mass Difference (ppm)	Possible Formula	Unsaturation Number
265.03528	63155.64	265.03570	-0.41	-1.56	$^{12}C_{13}{}^{1}H_{13}{}^{16}O_2{}^{32}S_2$	9.5

Figure S4. HRMS-DART$^+$ (19.8 eV) of **2**.

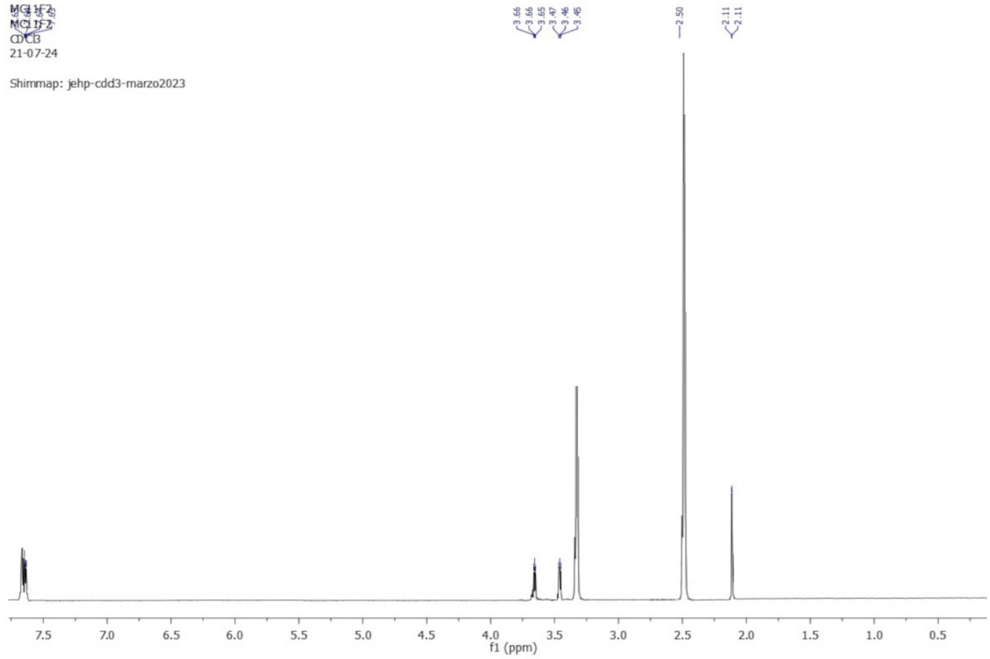

Figure S5. ^{1}H NMR (DMSO-d_6/TMS, 300 MHz) of **3**.

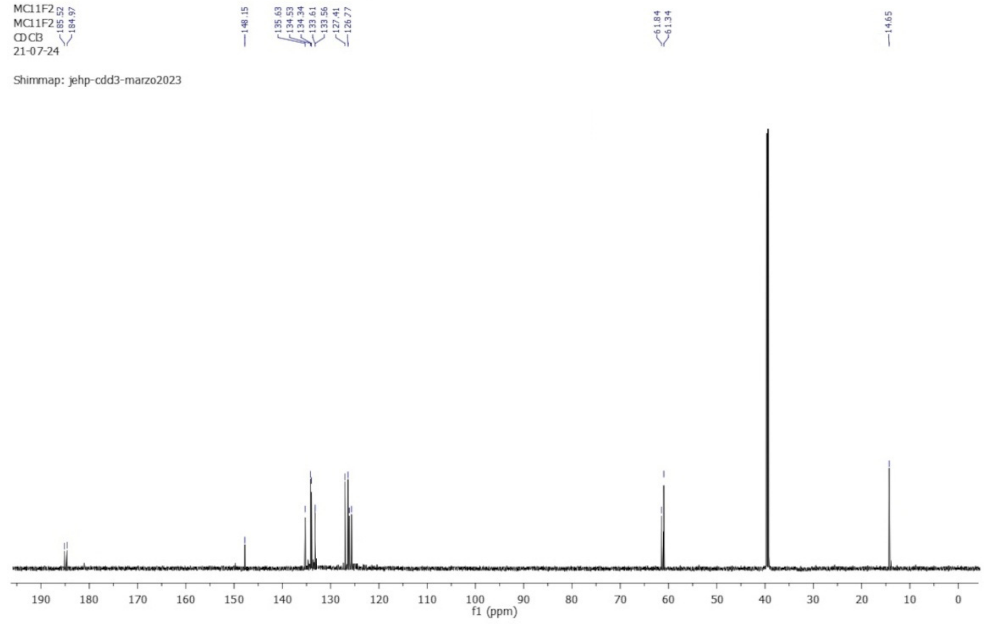

Figure S6. ^{13}C NMR (DMSO-d_6/TMS, 75 MHz) of **3**.

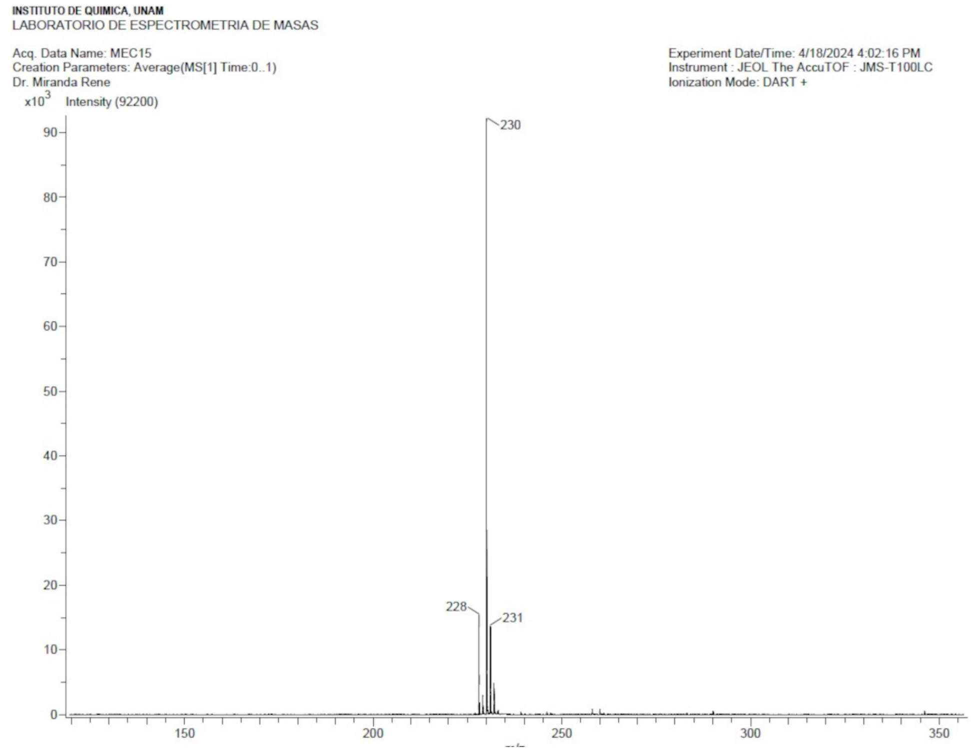

Figure S7. MS-DART$^+$ (19.8 eV) of **3**.

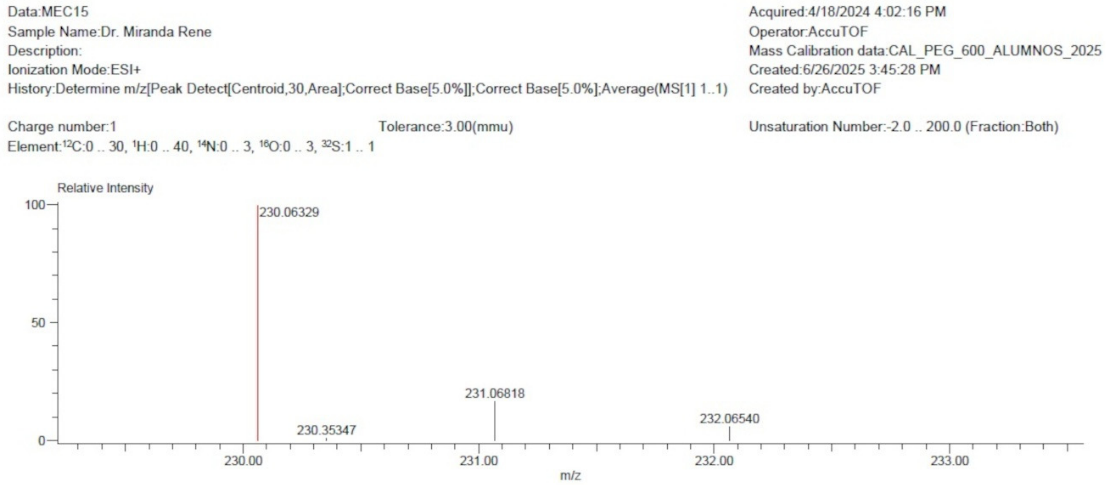

Mass	Intensity	Calc. Mass	Mass Difference (mmu)	Mass Difference (ppm)	Possible Formula	Unsaturation Number
230.06329	154437.67	230.06396	-0.67	-2.89	$^{12}C_{13}{}^{1}H_{12}{}^{14}N_{1}{}^{16}O_{1}{}^{32}S_{1}$	8.5

Figure S8. HRMS-DART$^+$ (19.8 eV) of **3**.

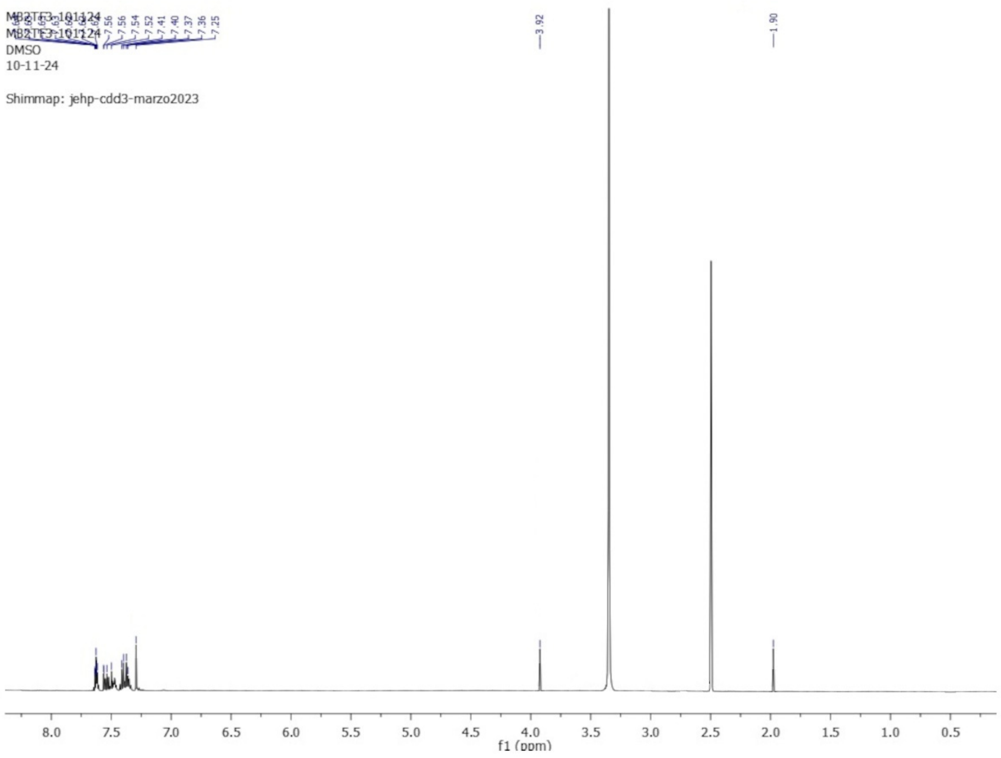

Figure S9. ^{1}H NMR (DMSO-d_6/TMS, 300 MHz) of **4**.

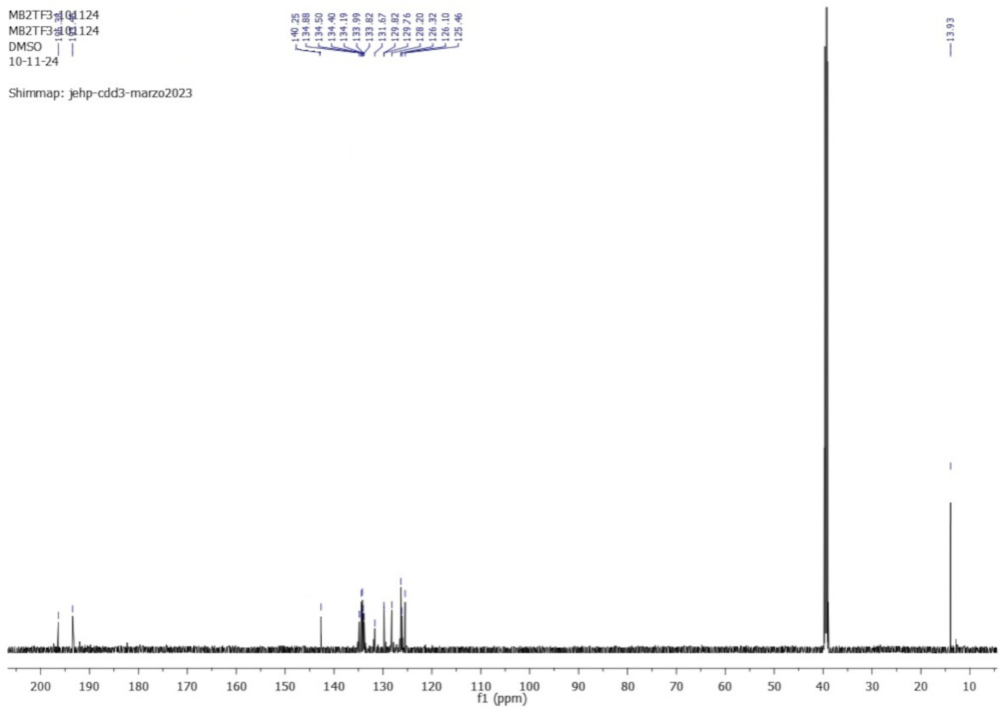

Figure S10. ^{13}C NMR (DMSO-d_6/TMS, 75 MHz) of **4**.

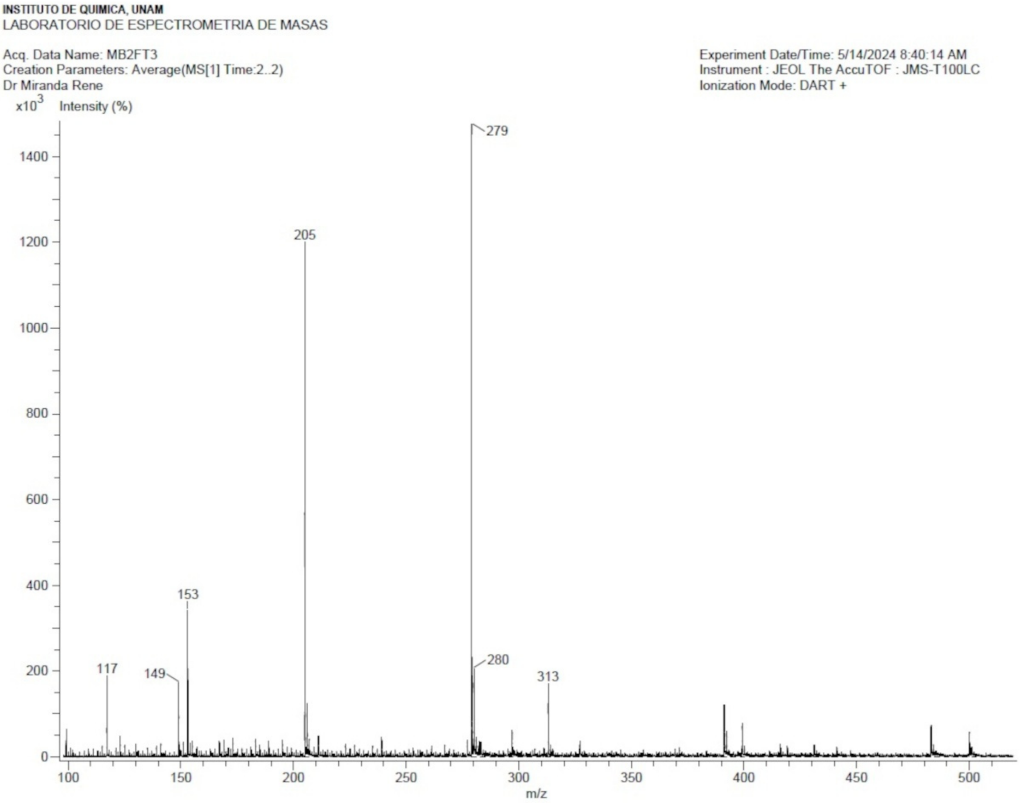

Figure S11. MS-DART$^+$ (19.8 eV) of **4**.

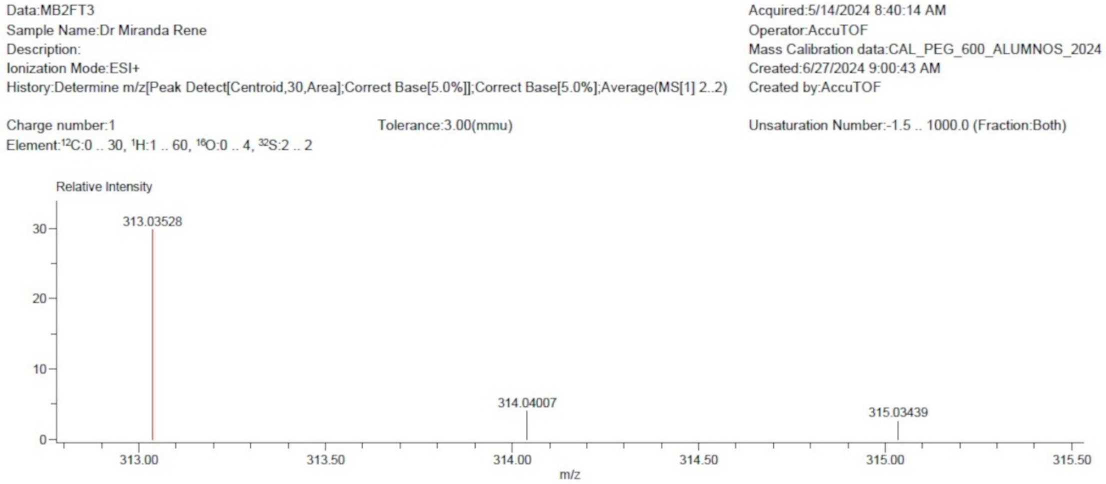

Mass	Intensity	Calc. Mass	Mass Difference (mmu)	Mass Difference (ppm)	Possible Formula	Unsaturation Number
313.03528	9979.77	313.03570	-0.41	-1.33	$^{12}C_{17}{}^{1}H_{13}{}^{16}O_2{}^{32}S_2$	13.5

Figure S12. HRMS-DART$^+$ (19.8 eV) of **4**.

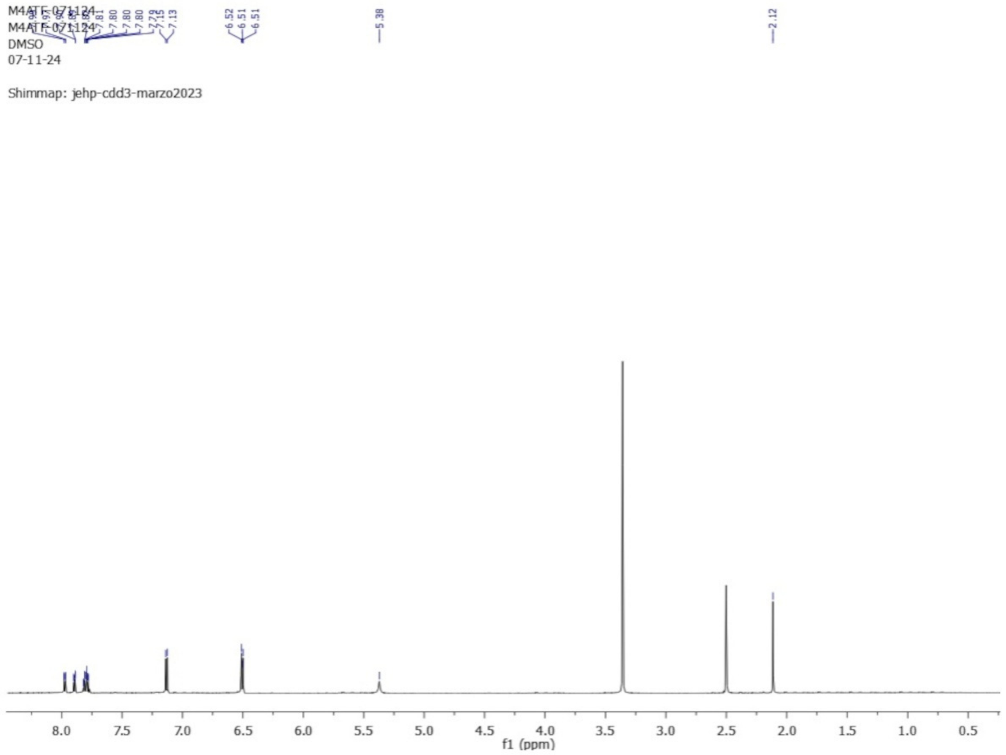

Figure S13. ^{1}H NMR (DMSO-d_6/TMS, 300 MHz) of **5**.

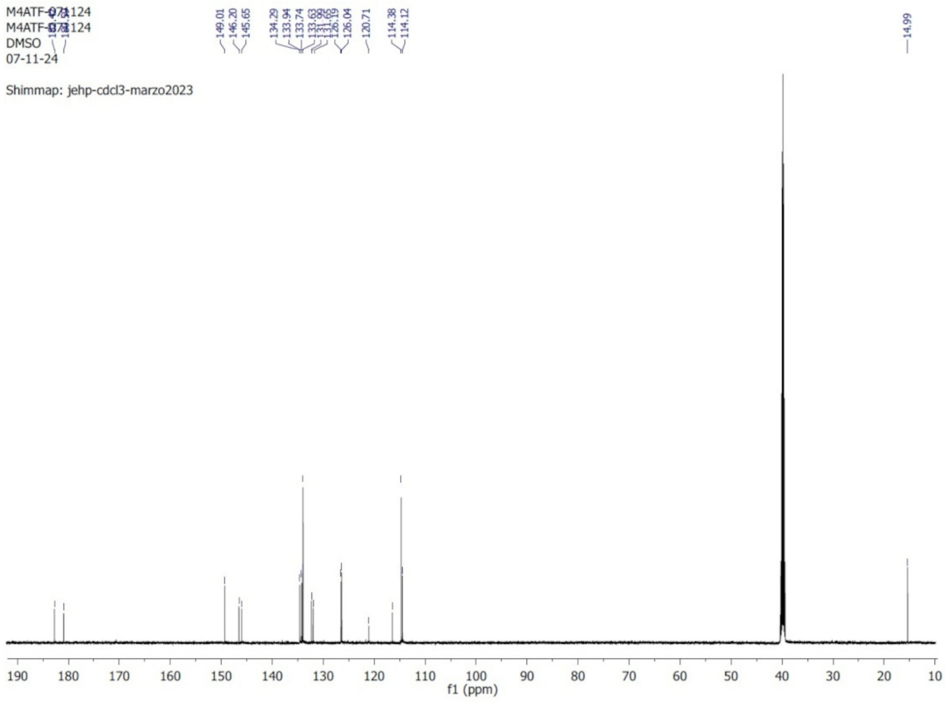

Figure S14. ^{13}C NMR (DMSO-d_6/TMS, 75 MHz) of **5**.

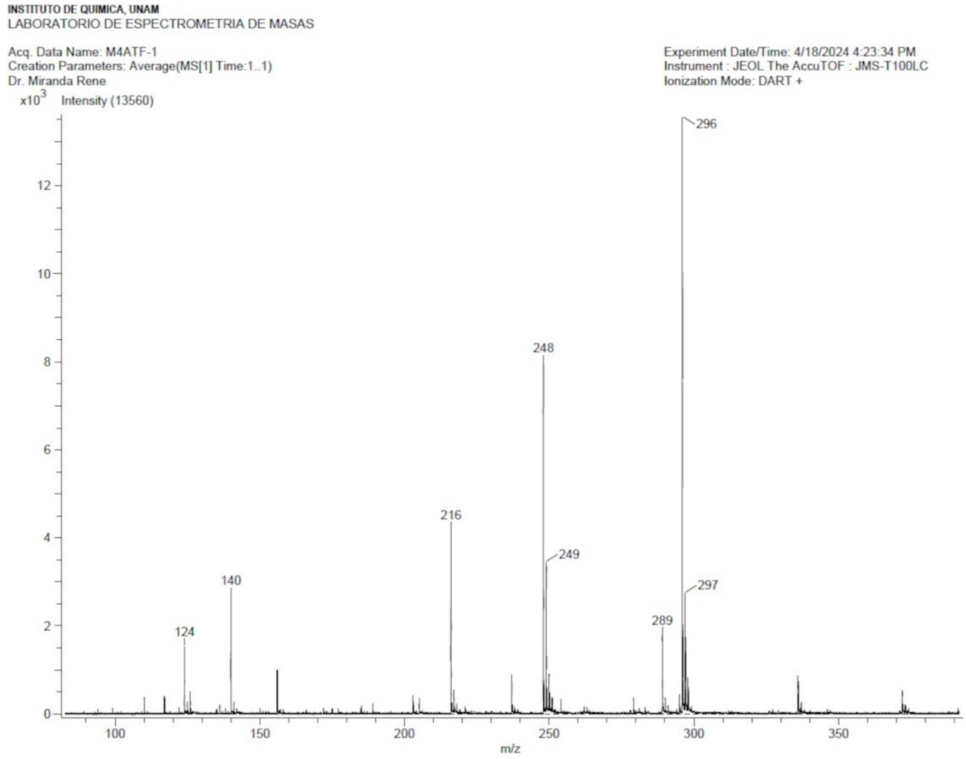

Figure S15. MS-DART$^+$ (19.8 eV) of **5**.

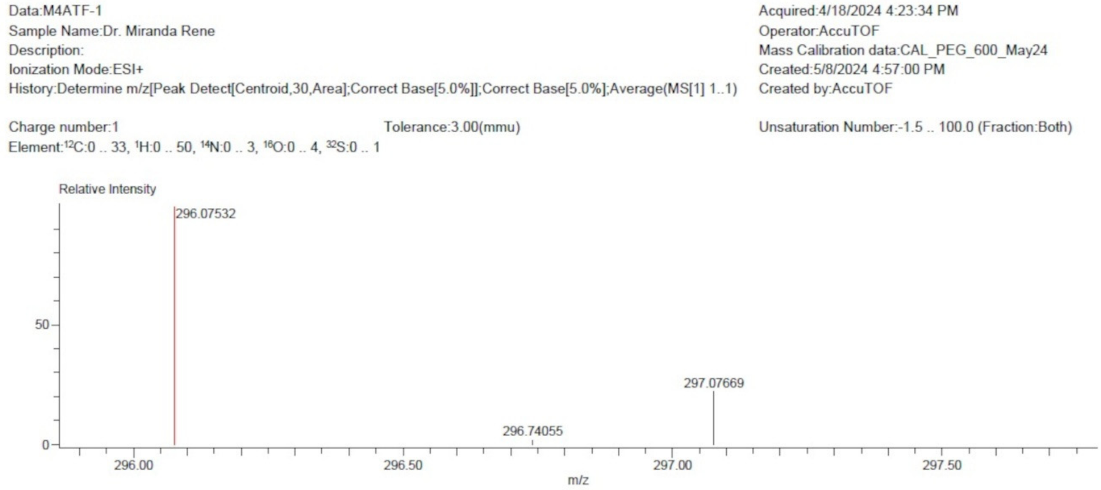

Mass	Intensity	Calc. Mass	Mass Difference (mmu)	Mass Difference (ppm)	Possible Formula	Unsaturation Number
296.07532	60366.11	296.07452	0.80	2.69	$^{12}C_{17}{}^{1}H_{14}{}^{14}N_{1}{}^{16}O_{2}{}^{32}S_{1}$	12.5

Figure S16. HRMS-DART$^+$ (19.8 eV) of **5**.
